# Explaining event-related fields by a mechanistic model encapsulating the anatomical structure of auditory cortex

**DOI:** 10.1007/s00422-019-00795-9

**Published:** 2019-02-28

**Authors:** Aida Hajizadeh, Artur Matysiak, Patrick J. C. May, Reinhard König

**Affiliations:** 1 Department of Psychology, Lancaster University, Lancaster, LA1 4YF UK; 2 Special Lab Non-invasive Brain Imaging, Leibniz Institute for Neurobiology, Brenneckestraße 6, 39118 Magdeburg, Germany

**Keywords:** Analytical solutions, Auditory cortex, Computational modeling, Event-related field, Event-related response, Magnetoencephalography, Normal modes

## Abstract

Event-related fields of the magnetoencephalogram are triggered by sensory stimuli and appear as a series of waves extending hundreds of milliseconds after stimulus onset. They reflect the processing of the stimulus in cortex and have a highly subject-specific morphology. However, we still have an incomplete picture of how event-related fields are generated, what the various waves signify, and why they are so subject-specific. Here, we focus on this problem through the lens of a computational model which describes auditory cortex in terms of interconnected cortical columns as part of hierarchically placed fields of the core, belt, and parabelt areas. We develop an analytical approach arriving at solutions to the system dynamics in terms of normal modes: damped harmonic oscillators emerging out of the coupled excitation and inhibition in the system. Each normal mode is a global feature which depends on the anatomical structure of the entire auditory cortex. Further, normal modes are fundamental dynamical building blocks, in that the activity of each cortical column represents a combination of all normal modes. This approach allows us to replicate a typical auditory event-related response as a weighted sum of the single-column activities. Our work offers an alternative to the view that the event-related field arises out of spatially discrete, local generators. Rather, there is only a single generator process distributed over the entire network of the auditory cortex. We present predictions for testing to what degree subject-specificity is due to cross-subject variations in dynamical parameters rather than in the cortical surface morphology.

## Introduction

The event-related potential (ERP) and field (ERF) measured with electroencephalography (EEG) and magnetoencephalography (MEG), respectively, appear as a series of waves triggered by a stimulus event. First described by Davis (1939), these waves are thought to represent stimulus-related activations which are stationary, time-locked to stimulus presentation, and buried in ongoing oscillations and other activity unrelated to stimulus processing. Thus, to cancel out the signal not associated with the stimulus, ERPs and ERFs are obtained through stimulus repetition and averaging of the single-trial EEG/MEG signals with respect to stimulus onset. The peaks and troughs of event-related responses function as landmarks as they can be identified in most subjects. Even so, the morphology of these responses varies greatly from subject to subject (see, for example, Atcherson et al. 2006; Dalebout and Robey 1997; Zacharias et al. 2011; Matysiak et al. 2013; König et al. 2015). Importantly, despite the straightforwardness of the method to extract ERPs and ERFs and decades of its use, and regardless of improvements in localization methods, we still have a poor understanding of how event-related responses are generated and what they signify.

In contrast, the general biophysics of EEG and MEG generation and the neural processes giving rise to currents in the brain contributing to these signals are well known (Sarvas 1987; Williamson and Kaufman 1981). EEG (Buzsáki et al. 2012; Einevoll et al. 2013; Mitzdorf 1985, 1994) and MEG signals (Hämäläinen et al. 1993; Okada et al. 1997) represent primarily a weighted sum of synchronized synaptic activities of pyramidal neural populations, whereas inhibitory neurons, with shorter dendrites and a symmetric dendritic structure, contribute to a closed field which does not show up in EEG and MEG. With pyramidal neurons being the predominant cell type in cortex, cortical columns are characterized by the apical dendrites of these cells running in parallel to each other and orthogonally to the cortical surface. The activity of *excitatory* synapses on these dendrites translates into electric current (cations $\text {Ca}^{2+}$ and $\text {Na}^{+}$) flowing into the apical dendrites, then along the dendrites as the primary/lead current, and out through the passive leak channels into the extracellular space, where the resulting volume current completes the circuit. The primary current along many synchronously activated pyramidal cells gives rise to a magnetic field which is visible in MEG and whose strength depends on the orientation and distance of the primary current in relation to the sensor. Similarly, the extracellular sinks and sources separated along the axis of the dendrites contribute to an open electric field which can be picked up in EEG and local field potential (LFP) measurements. Traditionally, *inhibitory* synapses onto pyramidal cells were thought to contribute only minimally to EEG/MEG, with the reversal potentials of these synapses being close to the resting membrane potential (Bartos et al. 2007; Mitzdorf 1985). Accordingly, an activated inhibitory synapse leads to minimal cross-membrane currents and hence a minimal contribution to EEG and MEG. However, when pyramidal neurons are spiking, for example, when spontaneous activity occurs, the membrane potential is elevated and so inhibitory synapses can significantly contribute to EEG and MEG generation (Trevelyan 2009; Glickfield et al. 2009; Bazelot et al. 2010).

The leap from biophysics to an understanding of the experimentally measured ERP and ERF waveforms is more difficult. A sensory stimulus (the event) sets off a series of neural activations propagating from the sensory organ to cortex. Cortical activations can be observed locally, in intracortical measurements, as increased spiking when, for example, the weak thalamocortical signal activates the local feedback circuits in cortical columns of the primary areas (Douglas et al. 1995), and this stimulus-evoked activation corresponds with the surface-recorded ERP (Shah et al. 2004). The auditory event-related response starts with small-amplitude, early-latency waves in the first 8 ms from stimulus onset; these are followed by mid-latency waves in the 8–40 ms range and, then, by large-amplitude, long-latency waves (e.g., Picton and Stuss 1980). In the passive recording condition, when the subject is not engaged in a task involving the stimuli, the most prominent waves of the auditory ERP are the long-latency P1, N1, and P2 responses, peaking at approximately 50, 100, and 200 ms, respectively. Their ERF counterparts are termed the P1m, N1m, and P2m.

Computational modeling can account for long-latency auditory ERPs purely in terms of interactions across cortical layers in primary auditory cortex (Wang and Knösche 2013). However, it seems unlikely that events in primary fields could represent the full intracortical counterpart of ERPs, which emerge as a superposition of activity across larger swathes of cortex. For example, in the case of auditory cortex (AC), anatomical studies in monkey show a hierarchical organization, with primary, core fields connecting to each other and to surrounding secondary, belt fields which, in turn, are connected with parabelt fields (Kaas and Hackett 2000; Hackett et al. 2014). There is physiological evidence to suggest that this hierarchical structure is reflected in feedforward activations progressing along the core–belt axis (Rauschecker 1997) and, hence, that cortical activations generating event-related responses should have temporal as well as spatial dynamics. This is supported by localization studies. Lütkenhöner and Steinsträter (1998) modeling the long-latency auditory ERF of a human subject with a single equivalent current dipole (ECD) found that the ECD location was non-stationary across the entire time course of the ERF: during the P1m, it lies on Heschls gyrus (HG) from where it slides to the planum temporale (PT) during the N1m and shifts back to HG during the P2m. Inui et al. (2006) performed multi-dipole analysis of auditory ERFs in a 120-ms post-stimulus time window using six ECDs located in the AC, and found that activity propagates along a roughly medial-lateral axis from HG to the superior temporal gyrus (STG). This was interpreted in terms of core–belt–parabelt activation. Similar results were reported by Yvert et al. (2005) who used minimum current estimates of recordings from intracerebral electrodes in human AC. Activity started in HG and Heschls sulcus (HS) at around 20 ms. The P1 time range (30–50 ms) was characterized by multiple areas becoming activated along medio-lateral and postero-anterior axes of propagation, successively involving HG and HS, PT, and STG. Subsequently, activity cycled back so that the rising slope of the N1 coincided with a similar series of activations as during the P1.

The above results point to event-related responses having both a temporal as well as a spatial dynamics whereby foci of activity in cortex shift over time. This addition of a spatial dimension to event-related responses adds to the descriptive palette but as such gives no deeper insight into what is going on, although there have been a number of approaches for gaining such insight. Research in the 1970s and 1980s posited that the event-related response is the linear sum of separable components, each generated by a spatially defined generator which also has a well-defined information processing function, such as stimulus onset detection or change detection (for reviews, see Näätänen and Picton 1987; Näätänen 1992). However, it has proven difficult to perform component separation in a reliable way (Lütkenhöner 1998) and to map components to anatomical structure (May and Tiitinen 2010). This emphasis on localization of activity was later complemented by considerations on the connections between cortical areas. In the framework of dynamical causal modeling (DCM), the event-related response is considered to arise out of a network of a small number of nodes arranged in a hierarchical structure and each representing an extended cortical area such as the primary or secondary AC (Friston et al. 2003; David et al. 2006). Stimulation-specific modulations in the response then arise out of changes in the strengths of connections, classified as bottom-up, lateral, or top-down. Such changes have been interpreted in the framework of predictive coding, whereby cortex attempts to predict incoming stimuli and in so doing generates prediction signals via top-down inhibitory connections. When there is a mismatch between stimulus and prediction, excitatory bottom-up connections relay a prediction error signal. In this view, the N1(m) signifies excitatory activity carrying the prediction error from AC toward frontal areas. In contrast, the P2(m) is due to inhibitory, feedback activity carrying the top-down prediction information (Garrido et al. 2007, 2009).

It appears then that we have a range of mutually exclusive explanations for event-related responses. First, these can be understood as arising purely locally, as the result of intra-laminar dynamics within primary areas (Wang and Knösche 2013). Second, they can be modeled as being generated by a single source with a continually shifting location (Lütkenhöner and Steinsträter 1998). Third, they can be seen to represent the linear sum of activity of a limited number of component generators, each performing an independent information processing task (Näätänen and Picton 1987). Fourth, they might arise out of a limited number of cortical areas interacting with each other in the performance of predictive coding (Friston et al. 2003). The spatial resolutions of these explanations seem to lie at the extremes, ranging from the single column to treating entire areas as single nodes (see also Ritter et al. 2013). None of these explanations are designed to represent transformations occurring within AC, because the internal dynamics of AC as a distributed system are not included. For this purpose, a more mechanistic view on how AC processes and represents sound is needed. Such a view could be based on the structure of the AC in order to account for the spatial dynamics occurring within the temporal lobe, as described above.

Thus, the purpose of the current study is to plug the resolution gap by bringing the anatomical structure of the AC into the explanation of the auditory event-related response. As a starting point we use a previously developed model of AC (May and Tiitinen 2010, 2013; May et al. 2015), and we restrict ourselves to examining ERF generation. The original model is highly nonlinear, and we simplify it in order to make an analytical approach possible. We derive analytical solutions to the model so as to characterize the dynamics of AC signal processing in terms of basic elements, so-called normal modes. This allows us then to address the following questions: How do ERFs originate from these dynamical elements? How do these elements depend on the anatomical core–belt–parabelt structure of the AC? And how is the ERF signal modulated by the topography of the primary currents, that is, by their orientation and distance from the MEG sensor? This analysis, then, lets us explore the origin of the subject-specificity of event-related responses: Why do subjects have unique ERF morphology? Can this be fully accounted for in terms of individual curvature of AC and its modulating effect on the MEG? Or do subjects also have unique dynamics of the auditory cortex?

## Model of auditory cortex

**Fig. 1:**
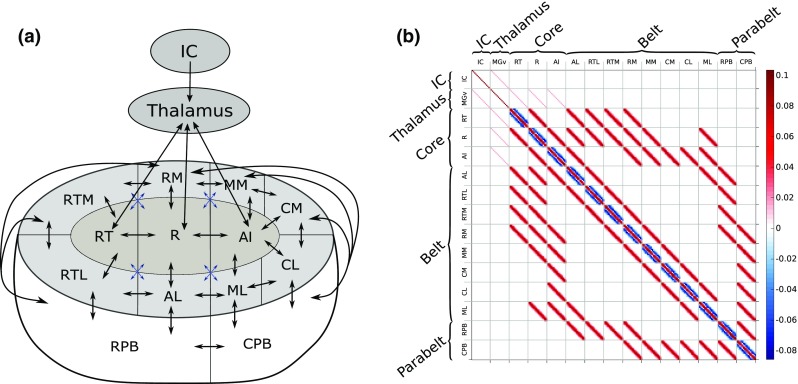
The anatomical structure of the model. **a** The organization of the model mimics that of the primate AC, being subdivided into 13 fields—three core, eight belt, and two parabelt fields (Baumann et al. 2013; Kaas and Hackett 2000; Hackett et al. 2014). The model also includes two subcortical fields representing inferior colliculus (IC) and thalamus. Each field contains 16 columns comprising interacting excitatory and inhibitory mean-field populations. Connections between fields are indicated by black and blue arrows which signify high and low density of connections, respectively. The cortical connections are bi-directional and result in multiple streams of feedforward and feedback activation. The input to AC is guided along direct connections from thalamus to the three core fields. **b** The connections between the fields are given expression in connection matrices where each element denotes the strength of the connection between two columns; here, the matrix $W_{{\mathrm{AC}}}$ is shown as an example. The values of the matrix elements indicated by the color bar to the right represent the strength of the connections between any two columns: positive values stand for excitatory, negative values for inhibitory connections

May and colleagues (May and Tiitinen 2010, 2013; May et al. 2015; Westö et al. 2016) developed a computational model of AC with anatomical structure and short-term plasticity of the synapses as central features, and with the aim of linking non-invasive results with in-vivo single- and multi-unit observations. The intuition behind this previous modeling work has been that auditory phenomena emerge from large-scale interactions in the auditory cortex. With a detailed map of the human AC still missing, the model borrows the core–belt–parabelt organization of the primate AC (Baumann et al. 2013; Hackett et al. 2014), as shown in Fig. 1a, with multiple streams of feedforward and feedback activation indicated by the arrows. The model replicates a wide range of temporal binding (across-time) phenomena observed in invasive experiments. These include forward masking (Brosch and Schreiner 1997; Brosch and Scheich 2008), stimulus-specific adaptation (Ulanovsky et al. 2003, 2004), two-tone facilitation (Brosch et al. 1999), and selective responses to complex sounds such as speech and monkey calls (Rauschecker 1997). Non-invasively observed phenomena explained by the model include the adaptation of the N1(m), and the emergence of the mismatch response as a dependence of the N1(m) on stimulus statistics (May and Tiitinen 2010; May et al. 2015). We note that by including the hierarchical structure of the whole AC at the cost of keeping the local dynamics relatively simple, this approach diverges from modeling efforts which describe primary AC only and concentrate on certain aspects of auditory processing, such as frequency tuning and forward masking (Loebel et al. 2007), stimulus-specific adaptation (Yarden and Nelken 2017), auditory induction (Noto et al. 2016), bird song discrimination (Larson et al. 2009), the N1/N1m response (Wang and Knösche 2013), or the mismatch response (May et al. 1999). Further, this approach is superficially similar to DCM in that both describe neural activations in terms of nodes in a hierarchically organized network. However, the DCM network is on a larger scale, extending across cortical lobes, and entire areas are compressed into single nodes. In contrast, the model of May and colleagues covers AC only, and it does so at a finer resolution, describing the organization of cortical columns in the various fields of the core, belt, and parabelt areas.

The dynamical unit of the model is a simplified description of the cortical column and it comprises two state variables *u* and *v* representing the population of excitatory (pyramidal) neurons and inhibitory interneurons, respectively. The dynamics are determined by the following two sets of coupled nonlinear differential equations (May and Tiitinen 2013; May et al. 2015):

1 $$\begin{aligned}&\begin{aligned} \tau _{\mathrm{m}}\dot{\mathbf{u }}(t)=&-\mathbf u (t) + A(t)\circ W_{\mathrm{ee}} \cdot g[\mathbf u (t)] \\&-\,W_{\mathrm{ei}}\cdot g[\mathbf v (t)] + \mathbf I _{\mathrm{aff,e}}(t) \end{aligned} \end{aligned}$$

2 $$\begin{aligned}&\begin{aligned} \tau _{\mathrm{m}}\dot{\mathbf{v }} (t)=&-\mathbf v (t) + W_{\mathrm{ie}} \cdot g[\mathbf u (t)] \\&-\,W_{\mathrm{ii}}\cdot g[\mathbf v (t)] + \mathbf {I}_{\mathrm{aff,i}}(t). \end{aligned} \end{aligned}$$

Here, $\tau _{\mathrm{m}}$ is the membrane time constant, $\mathbf u (t)=[u_1(t),\ldots ,u_N(t)]$ and $\mathbf v (t)=[v_1(t),\ldots ,v_N(t)]$ are the time-dependent vectors of the state variables, with $N = 240$, and $\circ$ denotes entrywise multiplication (Hadamard product). The subcortical afferent input vectors $\mathbf I _{\mathrm{aff,e}}(t)$ and $\mathbf I _{\mathrm{aff,i}}(t)$ target the excitatory (index ‘e’) and inhibitory (index ‘i’) cell populations of the three core fields, respectively, through 16 tonotopically organized frequency channels per field. The synaptic weights between populations are defined by the four connection matrices $W_{\mathrm{ee}}$, $W_{\mathrm{ei}}$, $W_{\mathrm{ie}}$, and $W_{\mathrm{ii}}$. We assume that all between-column connections are excitatory and encapsulated in the matrices $W_{\mathrm{ie}}$ and $W_{\mathrm{ee}}$; the latter also includes the long-range connections between fields. Further, we assume that $W_{\mathrm{ie}}$ has within-field elements only, and thus functional inhibition is of the lateral type. The matrices $W_{\mathrm{ei}}$ and $W_{\mathrm{ii}}$ have diagonal elements only and describe local, within-column inhibitory-to-excitatory and inhibitory-to-inhibitory connections. The output of each population is its mean spiking rate, which depends on the corresponding state variable through a continuous, nonlinear function *g*. Thus, the spiking rates $g[\mathbf u (t)]$ and $g[\mathbf v (t)]$ are zero for values of $\mathbf u (t)$ and $\mathbf v (t)$ smaller than a constant threshold $\theta$, and for values above this threshold they are monotonically increasing functions of the corresponding state variables, converging toward a saturation value of unity. The function *A*(*t*) is a time-varying matrix describing synaptic plasticity depending on pre-synaptic activity and governed by a differential equation of its own.

Equations (1) and (2) represent the mean-field leaky integrator neuron (LIN) model of classical neurodynamics as formulated by Hopfield (1984) and Hopfield and Tank (1986). The LIN model is related to the Wilson and Cowan (1972) model, which employs similar first-order differential equations to describe the interaction between neural populations, and where the state variables represent the proportion of neurons firing. The Hopfield-and-Tank formulation is slightly closer to the biologically realistic compartmental model, as the state variable can be seen as an approximation of the membrane potential whose time derivative depends on the cross-membrane currents. While originally intended as a single-unit description, the LIN model can be used as a population description by assuming that the units in the population are identically and symmetrically connected with each other, and that they all receive the same external input. In this case the population units behave identically with each other, and the population can be described by the unit equation. Because the equations refer to cross-membrane currents (i.e., synaptic and leak currents), it becomes easier to motivate the calculation of the MEG signal, as is discussed below. The LIN formulation also has the advantage that it opens up an analytical approach to the system dynamics.

Central to the model is the anatomical structure of AC (Fig. 1a). The AC organization is similar across mammals in the sense that a hierarchical core–belt–parabelt structure can be identified, although the number of fields and their connectivity with each other is species-specific (Budinger et al. 2000; Budinger and Heil 2005; Baumann et al. 2013; Hackett et al. 2014). In general, core fields are characterized by on-responses to pure tones and their preferential connections with the tonotopically organized division of the auditory thalamus. They have extensive local connections with each other and with the surrounding belt fields. Belt fields are also tonotopically organized, albeit with a lesser spatial frequency resolution. There are strong local connections of belt fields with core fields and neighboring parabelt fields as well as connections with other cortical areas. In addition to dense connections with the ventral division of the medial geniculate body, belt fields also have pronounced connections with non-tonotopic parts of the auditory thalamus. Parabelt fields are non-tonotopic and isocortical, with lower cell density than the belt fields and have connections mainly with non-tonotopic auditory and non-auditory thalamic nuclei and remote cortical areas.

The model mimics this structure with its 240 columns (32 subcortical, 208 cortical) being distributed into one field representing the inferior colliculus (IC), one thalamic field, three core fields, eight belt fields, and two parabelt fields, with each field comprising 16 columns. We note that the IC and thalamus were not part of the original model (May and Tiitinen 2013; May et al. 2015). As shown in Fig. 1a, the fields of the model are connected according to the scheme found in the macaque (Kaas and Hackett 2000). The connections between IC and thalamus as well as between thalamus and the three core fields are purely one-to-one tonotopic. The connections between cortical fields are likewise tonotopic, with each column projecting to its tonotopic counterpart in the recipient field. In addition, the projecting column also connects with columns neighboring the tonotopic counterpart. This spread of connections, which is symmetric and partly stochastic, is described by a Gaussian distribution and explained in more detail in Appendix A1. Hence, all the connections in the model are tonotopically organized, including those in the parabelt, and this simplification is unlikely to reflect the actual anatomical organization of AC. However, we note that the stochasticity in the connection matrix allows for the columns to exhibit multi-peaked and/or broad tuning curves.

## Modeling auditory cortex dynamics with normal modes

The previous modeling work in May et al. (2015) used numerical simulations of the nonlinear state equations, and accounted for the generation of the N1m and the mismatch response of the ERF. However, this approach gives only a snapshot of the system dynamics at the particular parameter settings chosen for the simulation. This way there is limited access to the relationship between the ERF on the one hand and the system parameters such as the synaptic weights and the anatomical organization on the other. Further, numerical simulations alone will not reveal why and when the peaks and troughs of the ERF occur. Here, we attempt to gain deeper insight into the dynamics of AC by taking the analytical approach to find solutions to the AC system dynamics by simplifying the description even further. In particular, we ignore synaptic plasticity, and we assume that the state variables inhabit the linear portion of the spiking-rate nonlinearity. We use this linearization of the spiking rate together with assumptions of symmetry of the weight matrices to decouple the two sets of state equations based on the standard approach of eigenvalue decomposition. The decoupled equations are then analytically solvable, and their solutions are referred to as normal modes of the system. We end up with a complete description of the system dynamics and the generated ERF in terms of the parameters of the AC. We note that synaptic plasticity will be addressed in future work and that its omission does not affect the validity of the current results.

The idea of cell populations operating in the quasi-linear range of the spiking-rate function was already used by Katznelson (1981) in his approach of decomposing cortical activity into spherical harmonics. Also, May and Tiitinen (2001) found that, with the assumption of a linear spiking rate, a pair of excitatory and inhibitory LINs can be described as a driven harmonic oscillator with damping. We therefore expect that linearization of the current AC model will likewise lead to oscillatory solutions, which can be considered to be the fundamental elements of cortical dynamics (Nunez 1995; Buzsáki and Draguhn 2004; Buzsáki 2006). These approximations gain some validity from the experimental observations of Allen et al. (1975) who found that neuronal responses behave linearly for a broad span of membrane potentials.

Our analytical approach is based on the original state equations for $\mathbf u (t)$ and $\mathbf v (t)$ outlined in Eqs. (1) and (2), where we have defined the linear spiking-rate functions $g(x) = \alpha x$ and we set $\alpha = 1$. In this formulation, negative spiking rates are possible, and they should be interpreted as values relative to a resting state of continuous, spontaneous firing. A standard approach to obtain analytical solutions of Eqs. (1) and (2) is to diagonalize this system of first-order constant-coefficient differential equations via eigenvalue decomposition. Given the oscillating nature of brain activity (e.g., Nunez 1995; Buzsáki and Draguhn 2004; Buzsáki 2006), we attempted to obtain a more intuitive understanding of ERFs in terms of damped harmonic oscillators. Thus, we realized the eigenvalue decomposition by first transforming Eqs. (1) and (2) into second-order differential equations (see Appendix A2):

3 $$\begin{aligned}&\ddot{\mathbf{u }}(t)+2\varGamma _u\dot{\mathbf{u }}(t)+\varOmega ^2_{0,u}{} \mathbf u (t)=\mathbf q (t) \end{aligned}$$

4 $$\begin{aligned}&\ddot{\mathbf{v }}(t)+2\varGamma _v\dot{\mathbf{v }}(t)+\varOmega ^2_{0,v}{} \mathbf v (t)=\mathbf j (t) \end{aligned}$$

Eqs. (3) and (4) describe the well-known physical problem of a system of $2 \times N$ driven damped coupled harmonic oscillators, each characterized by *N* degrees of freedom, and with the definitions

5 $$\begin{aligned} \varGamma _u= & {} \frac{\widetilde{W}_{\mathrm{ei}}\widetilde{W}_{\mathrm{ii}}\widetilde{W}_{\mathrm{ei}}^{-1}-\widetilde{W}_{\mathrm{ee}}}{2} \end{aligned}$$

6 $$\begin{aligned} \varGamma _v= & {} \frac{\widetilde{W}_{\mathrm{ii}}-\widetilde{W}_{\mathrm{ie}}\widetilde{W}_{\mathrm{ee}}\widetilde{W}_{\mathrm{ie}}^{-1}}{2} \end{aligned}$$

7 $$\begin{aligned} \varOmega ^2_{0,u}= & {} \widetilde{W}_{\mathrm{ei}}\widetilde{W}_{\mathrm{ie}}-\widetilde{W}_{\mathrm{ei}}\widetilde{W}_{\mathrm{ii}}\widetilde{W}_{\mathrm{ei}}^{-1}\widetilde{W}_{\mathrm{ee}} \end{aligned}$$

8 $$\begin{aligned} \varOmega ^2_{0,v}= & {} \widetilde{W}_{\mathrm{ie}}\widetilde{W}_{\mathrm{ei}}-\widetilde{W}_{\mathrm{ie}}\widetilde{W}_{\mathrm{ee}}\widetilde{W}_{\mathrm{ie}}^{-1}\widetilde{W}_{\mathrm{ii}} \end{aligned}$$

9 $$\begin{aligned} \mathbf q (t)= & {} \widetilde{W}_{\mathrm{ei}}\widetilde{W}_{\mathrm{ii}}\widetilde{W}_{\mathrm{ei}}^{-1}{} \mathbf I _{\mathrm{e}}(t)-\widetilde{W}_{\mathrm{ei}}\mathbf I _{\mathrm{i}}(t)+\dot{\mathbf{I }}_{\mathrm{e}}(t) \end{aligned}$$

10 $$\begin{aligned} \mathbf j (t)= & {} \widetilde{W}_{\mathrm{ie}}\mathbf I _{\mathrm{e}}(t)-\widetilde{W}_{\mathrm{ie}}\widetilde{W}_{\mathrm{ee}}\widetilde{W}_{\mathrm{ie}}^{-1}{} \mathbf I _{\mathrm{i}}(t)+\dot{\mathbf{I }}_{\mathrm{i}}(t). \end{aligned}$$

The matrix terms with the capping tilde represent the connection matrices of the linearized canonical form [see Eq. (26) in Appendix A2]. The matrices $\widetilde{W}_{\mathrm{ei}}$ and $\widetilde{W}_{\mathrm{ie}}$ are invertible because they are diagonal matrices.

**Fig. 2:**
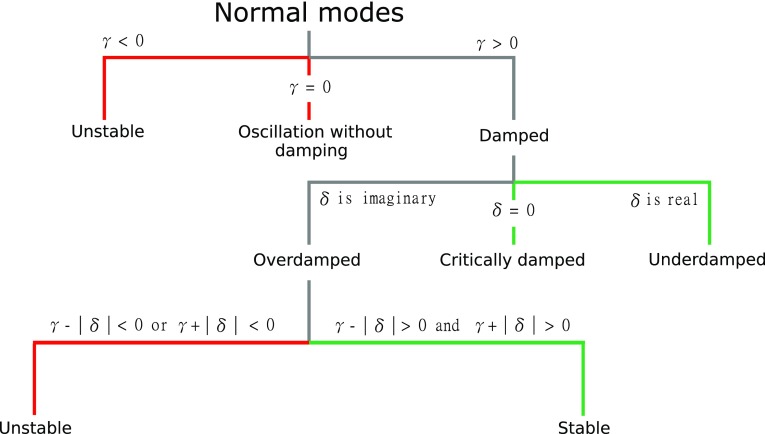
Flowchart illustrating how the stability of normal modes depends on the decay constant $\gamma$ and the damping frequency $\delta$. In general, stable states can only be reached for $\gamma > 0$, i.e., in damped systems. If in a given damped system the damping frequency is real-valued, the system is in a stable underdamped state; if $\delta$ equals to zero, the system is critically damped. For imaginary damping frequencies, the system is overdamped, in which case its stability depends on the sign of both the difference and sum of $\gamma$ and $\delta$

**Fig. 3:**
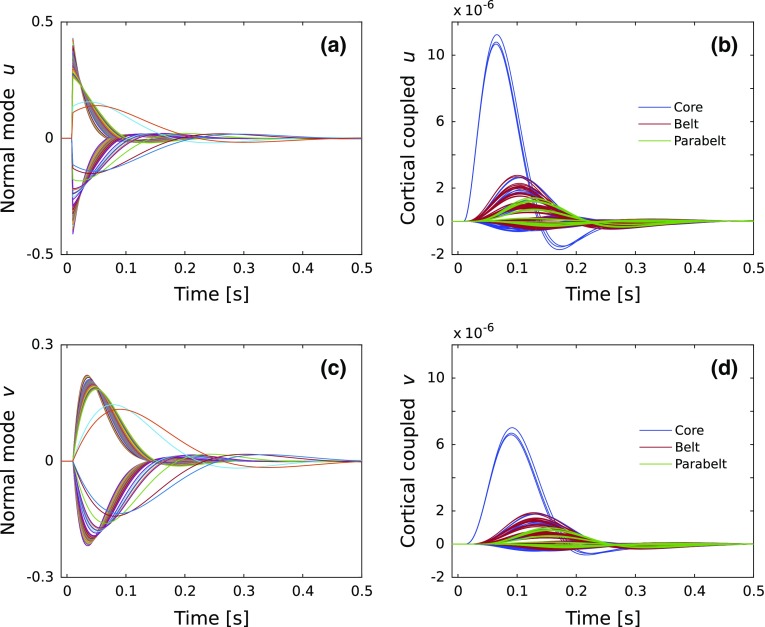
An example of the normalized normal modes and state variables for the excitatory (**a, b**) and inhibitory (**c, d**) cell populations. In this example, the normal modes are rapidly decaying underdamped oscillations. Only about one-third of the normal modes have a peak magnitude significantly different from zero; the majority of the modes decay from a tiny magnitude toward zero. Note that the normal modes shown in **a** and **c** cannot be attributed to individual cortical columns and instead represent system-wide elements of the dynamics of AC. The coupling of the normal modes according to Eq. (18) results in the waveforms of the state variables of the individual cortical columns. These waveforms, color-coded according to cortical area, are shown in **b** and **d**. The three dominant waveforms in blue represent the responses of the columns receiving the thalamic input in the three core fields. In comparison, the waveforms for the other core columns (blue), for the belt (red), and for the parabelt (green) have smaller peak magnitudes and larger peak latencies. The normal modes have been normalized such that the area covered by each waveform equals unity

Analytical solutions for $\mathbf u (t)$ and $\mathbf v (t)$ of Eqs. (3) and (4) can be found in the current eigenvalue approach if the coefficients $\varGamma _u$ and $\varOmega ^2_{0,u}$ as well as $\varGamma _v$ and $\varOmega ^2_{0,v}$ fulfill certain requirements (see Appendix A2). In this case, the complex system of *N* degrees of freedom is transformed into a representation where there are *N* decoupled oscillators, each with a single degree of freedom, and where the coefficients are diagonalized as indicated by the subscript ‘d’ [see Eqs. (47) and (48) in Appendix A2]:

11 $$\begin{aligned} \ddot{\mathbf{u }}_{\mathrm{d}}(t)+2 \varGamma _{\mathrm{d}}\dot{\mathbf{u }}_{\mathrm{d}}(t)+ \varOmega ^2_{0_{\mathrm{d}}}{} \mathbf u _{\mathrm{d}}(t)= & {} \mathbf q _{\mathrm{d}}(t) \end{aligned}$$

12 $$\begin{aligned} \ddot{\mathbf{v }}_{\mathrm{d}}(t)+2 \varGamma _{\mathrm{d}}\dot{\mathbf{v }}_{\mathrm{d}}(t)+ \varOmega ^2_{0_{\mathrm{d}}}{} \mathbf v _{\mathrm{d}}(t)= & {} \mathbf j _{\mathrm{d}}(t). \end{aligned}$$

**Fig. 4:**
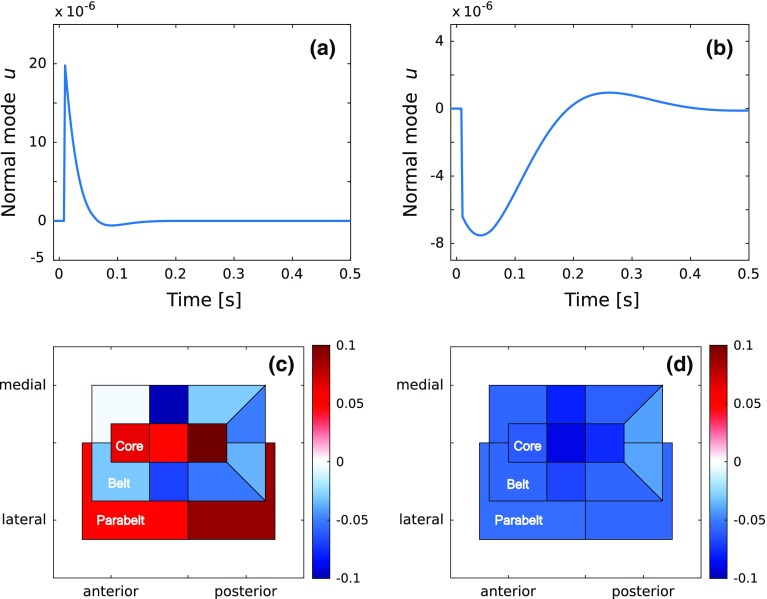
Mapping normal modes to anatomical structure. **a, b** An example of a normal mode with high damping frequency $\delta _{\mathrm{d}}$ and a normal mode with a low $\delta _{\mathrm{d}}$. The amplitude of the high-frequency normal mode dies away soon after 100 ms, whereas the low-frequency normal mode extends over 400 ms. **c, d** The normal modes shown in **a**, **b** are mapped onto anatomical structure (see Fig. 1a). For each normal mode, this mapping is achieved by identifying the elements in the mixing matrix $\varUpsilon$ corresponding to a particular cortical field and then averaging these elements. The high-frequency normal modes tend to result in high-structure mappings. In this example, the contributions of the normal mode to the three core fields and two parabelt fields are of opposite polarity to the contributions to the eight belt fields surrounding the core. The mapping of the low-frequency normal mode has less structure, with the polarity being the same across all fields

Each of the decoupled oscillators represents one normal mode with individual frequency and amplitude (Rayleigh 1945; Caughey 1960; Caughey and O’Kelly 1965). Normal modes are the basic elements of the decoupled system but they do not represent the dynamics of individual columns. Meaningful information on single-column dynamics as part of a network of columns can only be obtained after an inverse transformation whereby the normal modes are, in effect, coupled together.

The solutions for the decoupled equations for all $2\times 240$ normal modes of the two state variables in their vector representation are then:

13 $$\begin{aligned} u_{\mathrm{d}}(t)= & {} \exp (-\gamma _{\mathrm{d}} t)[a_{{u}_{\mathrm{d}}}\sin (\delta _{\mathrm{d}} t) + b_{{u}_{\mathrm{d}}}\cos (\delta _{\mathrm{d}} t)]+c_{{u}_{\mathrm{d}}} \end{aligned}$$

14 $$\begin{aligned} v_{\mathrm{d}}(t)= & {} \exp (-\gamma _{\mathrm{d}} t)[a_{{v}_{\mathrm{d}}}\sin (\delta _{\mathrm{d}} t) + b_{{v}_{\mathrm{d}}}\cos (\delta _{\mathrm{d}} t)]+c_{{v}_{\mathrm{d}}} , \end{aligned}$$

with the coefficients of $u_{\mathrm{d}}(t)$ given by

15 $$\begin{aligned} a_{{u}_{\mathrm{d}}}= & {} \frac{w_{\mathrm{ei}}\gamma _{\mathrm{d}}}{\omega ^2_{0_{\mathrm{d}}}\delta _{\mathrm{d}}} I_{\mathrm{i}}(t)+\frac{\omega ^2_{0_{\mathrm{d}}}+w_{\mathrm{ei}} w_{\mathrm{ie,d}}-w^2_{\mathrm{ii}}}{2\omega ^2_{0_{\mathrm{d}}}\delta _{\mathrm{d}}} I_{\mathrm{e}}(t)\nonumber \\&+\,\frac{w_{\mathrm{ii}}+w_{{\mathrm{AC,d}}}}{2\delta _{\mathrm{d}}}u_0 -\frac{w_{\mathrm{ie,d}}}{\delta _{\mathrm{d}}}v_0\nonumber \\ b_{{u}_{\mathrm{d}}}= & {} \frac{w_{\mathrm{ei}}}{\omega ^2_{0_{\mathrm{d}}}} I_{\mathrm{i}}(t)-\frac{w_{\mathrm{ii}}}{\omega ^2_{0_{\mathrm{d}}}}I_{\mathrm{e}}(t)+u_0\nonumber \\ c_{{u}_{\mathrm{d}}}= & {} -\frac{w_{\mathrm{ei}}}{\omega ^2_{0_{\mathrm{d}}}}I_{\mathrm{i}}(t) +\frac{w_{\mathrm{ii}}}{\omega ^2_{0_{\mathrm{d}}}}I_{\mathrm{e}}(t), \end{aligned}$$

and of $v_{\mathrm{d}}(t)$ given by

16 $$\begin{aligned} \begin{aligned} a_{{v}_{\mathrm{d}}} =&-\frac{w_{\mathrm{ie,d}}\gamma _{\mathrm{d}}}{\omega ^2_{0_{\mathrm{d}}} \delta _{\mathrm{d}}} I_{\mathrm{e}}(t)\\&+\,\frac{\omega ^2_{0_{\mathrm{d}}}+w_{\mathrm{ei}}w_{\mathrm{ie,d}}-w^2_{{\mathrm{AC,d}}}}{2\omega ^2_{0_{\mathrm{d}}}\delta _{\mathrm{d}}} I_{\mathrm{i}}(t)\\&-\,\frac{w_{\mathrm{ii}}+w_{{\mathrm{AC,d}}}}{2\delta _{\mathrm{d}}}v_0 +\frac{w_{\mathrm{ie,d}}}{\delta _{\mathrm{d}}}u_0\\ b_{{v}_{\mathrm{d}}}=&\frac{w_{{\mathrm{AC,d}}}}{\omega ^2_{0_{\mathrm{d}}}} I_{\mathrm{i}}(t)-\frac{w_{\mathrm{ie,d}}}{\omega ^2_{0_{\mathrm{d}}}} I_{\mathrm{e}}(t)+v_0\\ c_{{v}_{\mathrm{d}}}=&-\frac{w_{{\mathrm{AC,d}}}}{\omega ^2_{0_{\mathrm{d}}}} I_{\mathrm{i}}(t)+\frac{w_{\mathrm{ie,d}}}{\omega ^2_{0_{\mathrm{d}}}} I_{\mathrm{e}}(t). \end{aligned} \end{aligned}$$

The decay constant $\gamma _{\mathrm{d}}$, the angular frequency $\omega ^2_{0_{\mathrm{d}}}$, and the damping frequency $\delta _{\mathrm{d}}$ for both $u_{\mathrm{d}}(t)$ and $v_{\mathrm{d}}(t)$ depend on the connection matrices as follows:

17 $$\begin{aligned} \gamma _{\mathrm{d}}= & {} \frac{w_{\mathrm{ii}}-w_{{\mathrm{AC,d}}}}{2}\nonumber \\ \omega ^2_{0_{\mathrm{d}}}= & {} w_{\mathrm{ei}}w_{\mathrm{ie,d}}-w_{\mathrm{ii}}w_{{\mathrm{AC,d}}}\nonumber \\ \delta _{\mathrm{d}}= & {} \sqrt{\omega ^2_{0_{\mathrm{d}}}- \gamma ^2_{\mathrm{d}}}. \end{aligned}$$

The flowchart in Fig. 2 shows the general dependence of the normal modes on the values of $\gamma _{\mathrm{d}}$, $\omega ^2_{0_{\mathrm{d}}}$, and $\delta _{\mathrm{d}}$, and, thus, on the connection matrices. The sign of the decay constant $\gamma _{\mathrm{d}}$ determines whether the solutions of Eqs. (13) and (14) are unstable (for $\gamma _{\mathrm{d}} \le 0$) or whether they can be described in terms of a damped oscillator ($\gamma _{\mathrm{d}} > 0$). The value of the damping frequency $\delta _{\mathrm{d}}$ further classifies the damped normal modes into overdamped (imaginary-valued $\delta _{\mathrm{d}}$), critically damped ($\delta _{\mathrm{d}} = 0$) and underdamped (real-valued $\delta _{\mathrm{d}}$) solutions. The corresponding equations for these solutions are summarized in Appendix A2.

We now have simple mathematical expressions for describing the fundamental dynamics of the excitatory and inhibitory cell populations. Equations (13) and (14) represent the normal modes, the individual building blocks of the dynamics of the auditory cortex which depend on anatomical structure. Figure 3 shows an example of decoupled and coupled state variables when the model is presented with a 50-ms stimulus targeting the excitatory population of column 8 of the IC (amplitude = 0.01 for corresponding elements of $\mathbf I _{\mathrm{aff,e}}$). In Fig. 3a, c, the $2\times 240$ normal modes $u_{\mathrm{d}}(t)$ and $v_{\mathrm{d}}(t)$ are shown.

To gain access to the behavior of individual cortical columns, we have to couple the normal modes. This is achieved by multiplying the normal modes $\mathbf u _{\mathrm{d}}(t)$ with a mixing matrix $\varUpsilon$ according to

18 $$\begin{aligned} \mathbf u (t)=\varUpsilon \mathbf u _{\mathrm{d}}(t), \end{aligned}$$

and likewise for $\mathbf v (t)$. The columns of the matrix $\varUpsilon$ are the eigenvectors of $W_{{\mathrm{AC}}}$ (see explanations of Eqs. (41) and (42) in Appendix A2). As a consequence, the structure of the AC as a whole determines the coupled response of each column. Specifically, the mixing matrix $\varUpsilon$ determines how the normal modes are combined—uniquely for each column—to produce the coupled response. The results are the solutions for the state variables $\mathbf u (t)$ and $\mathbf v (t)$, and the corresponding waveforms are shown in Fig. 3b and 3d, respectively. For both the excitatory and inhibitory state variables, the waveforms of the core fields (blue curves) have the largest amplitude and smallest peak latency. The belt (red curves) and parabelt (green curves) waveforms are successively shifted to larger peak latencies, and their peak magnitudes are clearly smaller than those in the core.

Note that the assignment of any normal mode to a particular location in the AC is not possible. Instead, one can consider how each normal mode contributes to the activity of each column and field. Figure 4 shows two examples of normal modes, the first with a high damping frequency $\delta _{\mathrm{d}}$ (Fig. 4a) and the second with a low $\delta _{\mathrm{d}}$ and a polarity opposite to that of the first (Fig. 4b). Figure 4c, d shows how these normal modes are mapped onto the structure of the AC (see Fig. 1) in terms of their contributions averaged over each cortical field. Note that the maps represent the mean contribution of each normal mode to the activities of the individual fields. Specifically, this occurs through the multiplication of the mixing matrix with the normal mode [see Eq. (18)]. Figure 4 represents the general observation that the AC mappings of high-frequency normal modes tend to have more structure than those of low-frequency normal modes.

## A new framework for understanding ERF generation

The MEG signal arises out of the primary currents of synchronously active populations of pyramidal cells in cortex, and it is modified by topographical factors such as the current orientation and the distance to the MEG sensor. In this section, we describe how we approximate the primary currents, given that our model treats the neural populations as dimensionless dynamical units without an explicit term for dendritic current. We delineate how topographical factors can be included into the description, and how this produces two separate sets of parameters: one pertaining to the model dynamics and the other to topographical factors. We then demonstrate how the model replicates the ERF and what this reveals about ERF generation. Finally, we consider the cross-subject variability of the ERF with an eye to determining experimentally whether subject-specific responses are due to topographical factors only, or whether subject-specific cortical dynamics is also at play.

**Fig. 5:**
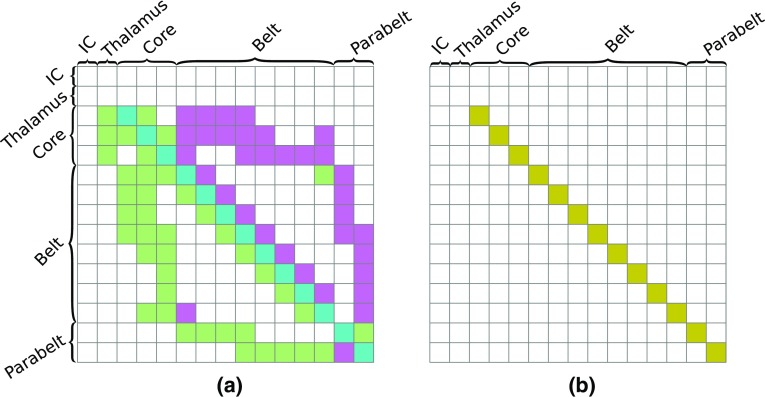
The structure of the $K_{{i}}$ matrices. The topographical modulations of the MEG signal are displayed as $15 \times 15$ field matrices, where each element represents $16 \times 16$ connections. The MEG signal is determined by the primary currents of the excitatory (pyramidal) cell populations and is thus driven by the excitatory and inhibitory inputs to these populations. **a** The matrix $K_{{\mathrm{1}}}$ modulates the contribution to the MEG made via the excitatory connections. The 88 nonzero entries of this field matrix comprise positive and negative elements representing feedback (purple) and feedforward (light green) connections. The elements on the main diagonal represent within-field connections (cyan). **b**$K_{{\mathrm{2}}}$ and $K_{{\mathrm{3}}}$ modulate the contribution to the MEG signal made by the intra-column and lateral inhibitory connections, respectively. Only 13 within-field elements have nonzero elements because we are assuming that all inhibition is local, originating within the same field

The MEG signal is proportional to the weighted sum of the primary currents, in the main those running in the apical dendrites of pyramidal neurons. While our description lacks a term for dendritic current, it does have terms equivalent to the synaptic input from each incoming connection. These are the spiking rates of the pre-synaptic populations multiplied by the connection strength. We approximated the primary current of each column to be directly proportional to the synaptic inputs to the excitatory populations. This approximation is justified by theoretical considerations and simulation results (May 2002) using realistic neuron models in the NEURON simulation environment (Hines and Carnevale 2001). These previous results show that the synaptic input current has a near-linear relationship with the axial current in the dendrite and therefore with the contribution to the MEG signal produced by the neuron. In our model, the synaptic input term depends directly on the pre-connection state variable and, thus, the dynamical element of the MEG signal is found in the solutions to the state variables, see Eqs. (13)–(17). The dynamical element of the MEG is therefore governed by the set of system parameters *P* for the dynamical equations, with *P* comprising the elements of the various connection matrices, the time constant $\tau _{\mathrm{m}}$, and the input–output function $g(\cdot )$.

**Table 1:** Default dynamical and topographical parameter values used in the simulations

Dynamical parameter set *P*	Value	Topographical parameter set $K_{{i}}$	Value
IC recurrent connections	0.09	$K_{{\mathrm{1}}}$ (feedforward)	$-$ 4
IC to Thalamus connections	0.015	$K_{{\mathrm{1}}}$ (feedback)	20
Thalamus recurrent connections	0.09	$K_{{\mathrm{1}}}$ (within field)	$-$ 5
Thalamus to core connections	0.015	$K_{{\mathrm{2}}}$ (within field)	2
$W_{\mathrm{AC}}$	See Table 2	$K_{{\mathrm{3}}}$ (within field)	2
$W_{\mathrm{ie,d}}$	1		
$W_{\mathrm{ei}}$	1		
$W_{\mathrm{ii}}$	0.2		
$\tau _{\mathrm{m}}$	40 ms		

The MEG is not just a linear aggregate of the magnitude of the primary currents. Rather, in the aggregation, the contribution from each primary current is modulated by non-dynamical, topographical factors: the distance of the current to the MEG sensor, the geometry of the volume conductor, and the orientation of the current (Hämäläinen et al. 1993). The latter depends on multiple aspects: whether the synapse driving the current is excitatory or inhibitory, the location of the synapse on the apical dendrite, and the orientation of the pyramidal cell containing the current—which, in turn, depends on the subject-specific topography of the cortical surface. Thus, the contribution to the primary current of each synapse has a unique topographical multiplier. Translating this into the context of our model, the topographical modulation of the MEG signal can be represented by a set of matrices $K_i$, one for each connection matrix. Thus, the expression for the MEG signal is a sum over the synaptic inputs to the excitatory populations, weighted by the $K_i$ matrices:

19 $$\begin{aligned} \begin{aligned} R(t)=&\sum _{ij}[K_{{{\mathrm{1}}}}[i,j] \circ W^{+}_{{{\mathrm{AC}}}} [i,j]u_j(t) \\&+\,K_{{{\mathrm{2}}}}[i,j] \circ W_{{{\mathrm{ei}}}}[i,j]v_j(t) \\&+\,K_{{{\mathrm{3}}}}[i,j] \circ W^{-}_{{{\mathrm{AC}}}}[i,j]u_j(t)]. \end{aligned} \end{aligned}$$

Here, the symbol $\circ$ represents the entrywise matrix multiplication, and the indices *i* and *j* refer to post- and pre-synaptic populations, respectively. The matrices $W^{+}_{{{\mathrm{AC}}}}$ and $W^{-}_{{{\mathrm{AC}}}}$ represent the excitatory connections and lateral inhibition of $W_{{\mathrm{AC}}}$, respectively.

**Fig. 6:**
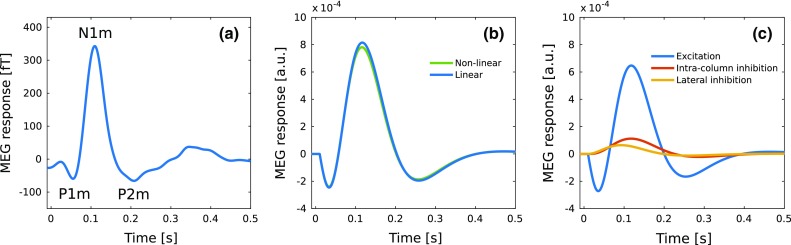
Comparison of experimental and simulated ERFs. **a** The figure shows the grand mean of trial-averaged ERFs recorded from 15 subjects in response to 1.5-kHz tones of 100-ms duration and 80-dB sensation level presented with a stimulus-onset interval of 3 s (data from Matysiak et al. 2013). **b** The blue curve represents the ERF computed according to Eq. (19) as the sum of the primary currents modulated by the three *K*-matrices. The simulation replicates a typical ERF waveform with P1m, N1m, and P2m responses. The green curve shows the simulated ERF generated by the nonlinear model with sigmoid spiking-rate function, synaptic plasticity, but otherwise model parameters identical to those used in the analytical approach. For more information on synaptic plasticity, see May et al. (2015). In **c**, the MEG signal resulting from the analytical solutions shown in **b** is broken down into the contributions made by the excitatory connections (blue), the intra-column inhibitory connections (red), and the connections for lateral inhibition (orange). Only the contribution from the excitatory connections shows distinct P1m, N1m, and P2m deflections, which have peak latencies and amplitudes similar to those of the overall ERF shown in **b**. In comparison, the contributions from the inhibitory connections are unimodal, with earlier peak latencies and much smaller peak amplitudes. Thus, the P1m and the P2m deflections are driven by excitatory connections. The origin of the abscissa ($t = 0$) indicates stimulus onset

### Calculating the MEG signal

In the sum of Eq. (19), the first term describes the contribution of the excitatory connections. The topographical information is embedded in the matrix $K_{{\mathrm{1}}}$ as shown in Fig. 5a. The elements of $K_{{\mathrm{1}}}$ represent three types of connection: feedforward, feedback, and within field (Table 1). Feedforward connections (light green in Fig. 5a) convey signals along the core–belt–parabelt direction. They contribute to the MEG with a polarity opposite to that of feedback connections (purple in Fig. 5a), which carry signals from the parabelt toward the core. This polarity reversal models the findings that feedforward signals tend to arrive in the middle layers, predominantly in layer IV, target proximal locations of the pyramidal dendrites, and thus result in a current flow which points upward, toward the cortical surface. In contrast, feedback signals result in a current flow downward because they arrive in upper layers I and II, and therefore target distal locations on the apical dendrite (Ahlfors et al. 2015). The feedforward–feedback organization of $K_{{\mathrm{1}}}$ is in part supported by the results of Hackett et al. (2014) who mapped the layer-specific feedforward and feedback projections in belt and parabelt of the monkey. The polarity of the within-field connections (cyan elements in Fig. 5a) was taken to be the same as for the feedforward connections.

**Fig. 7:**
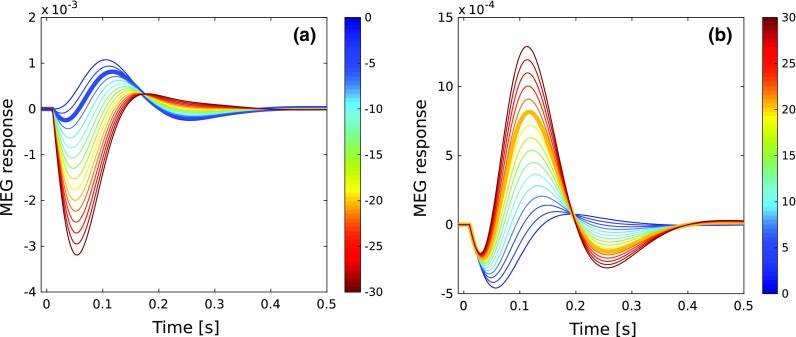
The role of feedforward and feedback connections in shaping the ERF. **a** The contribution of the feedforward connections as specified in $K_{{\mathrm{1}}}$ was varied from 0 (blue) to $-$ 30 (red) in steps of 2. All other parameters were fixed at their default values. With no feedforward contribution, the ERF comprises an N1m and P2m. As the feedforward term is increased, the P1m increases in prominence until it becomes the sole feature. **b** The contribution of the feedback connections was increased from zero (blue) to 30 (red) while the other parameters were kept at their default values. With no feedback contribution, the ERF contains a P1m response only. As the feedback contribution grows, the most noticeable change in the ERF is the emergence and increase of the N1m and P2m deflections. The thick blue curve in **a** and the thick yellow curve in **b** correspond to the simulated waveform shown in Fig. 6b

The second term of Eq. (19) is the contribution to the MEG of the inhibitory projections originating from within the column. The synaptic input is modulated by the matrix $K_{{\mathrm{2}}}$, as represented in Fig. 5b. The third term of Eq. (19) accounts for the MEG contribution of lateral inhibition whereby $W^{-}_{{\mathrm{AC}}}$ is modulated by the matrix $K_{{\mathrm{3}}}$. $K_{{\mathrm{2}}}$ and $K_{{\mathrm{3}}}$ have the same structure and values (Table 1). Further, the polarity of the elements of $K_{{\mathrm{2}}}$ and $K_{{\mathrm{3}}}$ is the same as that of the elements of $K_{{\mathrm{1}}}$ representing feedback connections. This polarity conveys the finding that inhibitory synapses tend to be located near the soma (Douglas et al. 2004, see, however, Kubota et al. 2016), and therefore their activation contributes to a current pointing downward in the apical dendrites of pyramidal neurons (Ahlfors and Wreh 2015). In general, inhibitory synapses contribute to the primary current if the pyramidal cell has an elevated membrane potential and is spiking (Trevelyan 2009; Glickfield et al. 2009; Bazelot et al. 2010). In our case, we are assuming that the resting state represents sustained, spontaneous activity, which corresponds to an elevated membrane potential.

### Simulations of ERFs

In each simulation, the AC model started in the resting state and was presented with a 50-ms stimulus, with the afferent input $\mathbf I _{\mathrm{aff,e}}$ targeting the central column of the IC field (column 8) with amplitude = 0.01. We used a low amplitude to ensure a good correspondence between the ERFs produced by the linear and nonlinear versions of the model. A 10-ms delay—roughly the duration of auditory brain stem responses—was assumed to occur between stimulus onset and the signal reaching IC. The default values of the dynamical parameters *P* and topographical parameters $K_i$ are listed in Table 1. The parameter values reflect the finding that recurrent connections in cortex are an order of magnitude stronger than afferent and between-field connections (Douglas and Martin 2007; Douglas et al. 1995). However, the exact values, while representing a balance between excitation and inhibition, are arbitrary and were chosen on the basis of reproducing realistic looking ERFs. The ERF was calculated using Eq. (19).

**Fig. 8:**
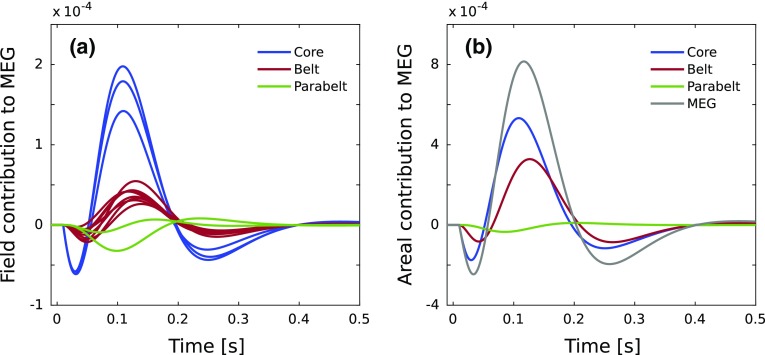
Contributions from AC fields and areas to the ERF. **a** The contributions of the individual AC fields decrease sharply as one moves from core to belt to parabelt. **b** The larger number of belt fields (eight) compared to those of the core (three) compensates for this disparity such that the total contribution of the belt to the overall ERF is of the same order of magnitude as that of the core. The magnitude of the parabelt response is much weaker. For comparison, the overall ERF waveform (see Fig. 6b) is also displayed (gray line)

**Fig. 9:**
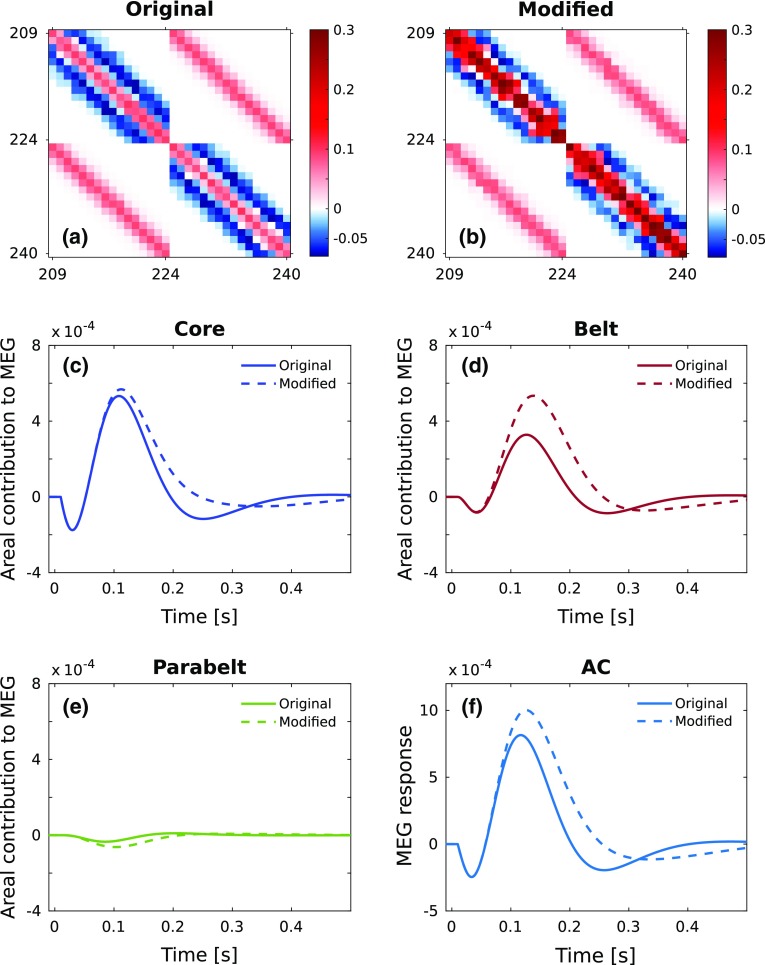
Evoked responses are network properties as demonstrated by modulating the connections within the parabelt. **a** The original connections within and between the two parabelt fields are shown as a magnification of $W_{{\mathrm{AC}}}$ of Fig. 1b. **b** The weights of the within-field connections in parabelt were modified such that the default value of the amplitude *r* of the Gaussian distribution was multiplied by 3. Further, the default values of the stochasticity parameter *s* for both the within- and between-field connections in parabelt were multiplied by 2 (see Table 1 and Appendix A1). The color bars indicate the connection strength and polarity. **c**–**e** The contributions to the ERF are shown separately for the core, belt and parabelt. The solid curves represent simulations using the original parabelt connections, and they are identical to those shown in Fig. 8b. The dashed curves are from simulations using the parabelt-modified $W_{{\mathrm{AC}}}$ shown in **b**. The modification of the parabelt connections affects the ERF contributions from all areas, including the core. **f** This results in marked changes in the morphology of the ERF, including shifts in the peak latency and amplitude of the N1m and the abolition of the P2m

Figure 6a shows an example of an ERF from an MEG experiment using pure-tone stimulation (Matysiak et al. 2013). The waveform has a typical morphology and it shows the grand mean computed from the ERFs of several subjects. The blue curve in Fig. 6b represents the ERF waveform generated by the current linear model whose normal modes and coupled state variables are presented in Fig. 3. This simulation replicates the morphology shown for the experimental ERF in Fig. 6a: There is an initial P1m-like response peaking at 35 ms. The ERF then crosses polarity and builds up into a large-amplitude N1m-like response peaking at 115 ms. This then is followed by a shallow P2m response peaking at 260 ms. The green curve was generated in a simulation with the nonlinear version of the model. The linear and nonlinear models produce ERFs with very similar morphologies. The minute differences in the peak amplitudes are caused by the synaptic plasticity term of the nonlinear model.

Figure 6c shows the contributions to the ERF coming from excitation, intra-column inhibition, and lateral inhibition, as defined in Eq. (19). The contribution from excitation is characterized by deflections similar to the P1m, N1m, and P2m responses (Fig. 6b). In contrast, the contributions made via the inhibitory connections each comprise only a single N1m-like deflection of a small amplitude. Thus, the P1m and P2m responses are driven by excitation of the pyramidal cells.

To further examine the dependence of the ERF on the topography of the connections, we varied the $K_{{{\mathrm{1}}}}$ weights of the contributions of the feedforward and feedback connections to the MEG signal. The results are shown in Fig. 7, where the thick blue line in (a) and the thick yellow line in (b) depict the waveform in Fig. 6b generated with the default parameter values (Table 1). In Fig. 7a, the contribution of the feedforward connections (light green elements in Fig. 5a; default value $-$ 4) was altered from 0 (top blue line) to $-\,30$ (bottom red line) in steps of 2 while keeping the other parameters constant. The largest effect is the emergence of the P1m and a marked monotonic increase in its peak amplitude as the feedforward contribution is increased. This is accompanied by an increase of the P1m peak latency from 26 to 54 ms. As the P1m becomes more and more substantial, it increasingly dwarfs the N1m, which is abolished at the largest contributions of the feedforward connections. A very different pattern emerged when the feedback contribution to the MEG signal (purple elements in Fig. 5a; default value 20) was increased from 0 (bottom blue line) to 30 (top red line) in steps of 2 (Fig. 7b). With zero feedback contribution, the N1m and the P2m were missing, and the ERF comprised a P1m response only. The N1m and P2m emerged only with the presence of feedback contribution. As this contribution was increased, the largest growth in amplitude was for the N1m. These results suggest that the P1m mainly reflects feedforward activation, whereas the N1m and P2m reflect feedback activation.

In Fig. 8a, the source structure of the ERF shown in Fig. 6b is revealed in terms of the individual contributions from the 13 cortical fields. The total contributions from the core, belt and parabelt are shown in Fig. 8b, along with the overall MEG response. In general, as one moves along the core–belt–parabelt axis, the responses decrease in magnitude and increase in latency. The P1m has its main source in the core, with the belt also contributing. Similarly, the N1m is largely generated in the core, but the belt contribution is now much larger. The core and belt have similar contributions to the P2m. Because of their delay, the parabelt responses contribute to the ERFs with deflections of the opposite polarity of those produced by the core and belt. However, these contributions are very shallow and broad. We note that none of the peaks and troughs of the ERF (e.g., the P1m, N1m, P2m) has a dedicated response generator in the sense that activity in any particular region of the model would account for the deflection. Rather, activity is occurring in all parts of the AC throughout the ERF, with the exception of the parabelt being in its resting state during the P1m. What is changing between the ERF deflections is the relative contribution of each area to the signal.

Each field and area might play a more fundamental role in ERF generation than that of providing a source for each deflection. Namely, our analytical results in Eqs. (13)–(17) show that the anatomical structure of the entire AC, encapsulated in $W_{{\mathrm{AC}}}$, is part of the solution to the dynamical equations. Thus, for each field, the way it is connected to other fields, and even the local structure within the field should impact on the entire ERF. This should be the case even at the fringe of the model, in the parabelt, which otherwise provides only a weak direct source to the ERF. Figure 9 shows the results of the simulations testing this idea. Here, while keeping all other parameters constant, we introduced variations to the weight values of $W_{{\mathrm{AC}}}$ representing the internal connections within and between the two parabelt fields (for details, see Appendix A1). These variations (Fig. 9a, b) had a minimal effect on the response produced by the parabelt (Fig. 9e) while, paradoxically, significantly altering the overall ERF (Fig. 9f). Figure 9c, d shows that the parabelt modification resulted in prominent changes in the core and belt contributions to the N1m. The end result in the ERF is a much broader N1m waveform, with a larger peak amplitude and latency, and an elimination of the P2m.

### Separating dynamics from topography in ERF generation

**Fig. 10:**
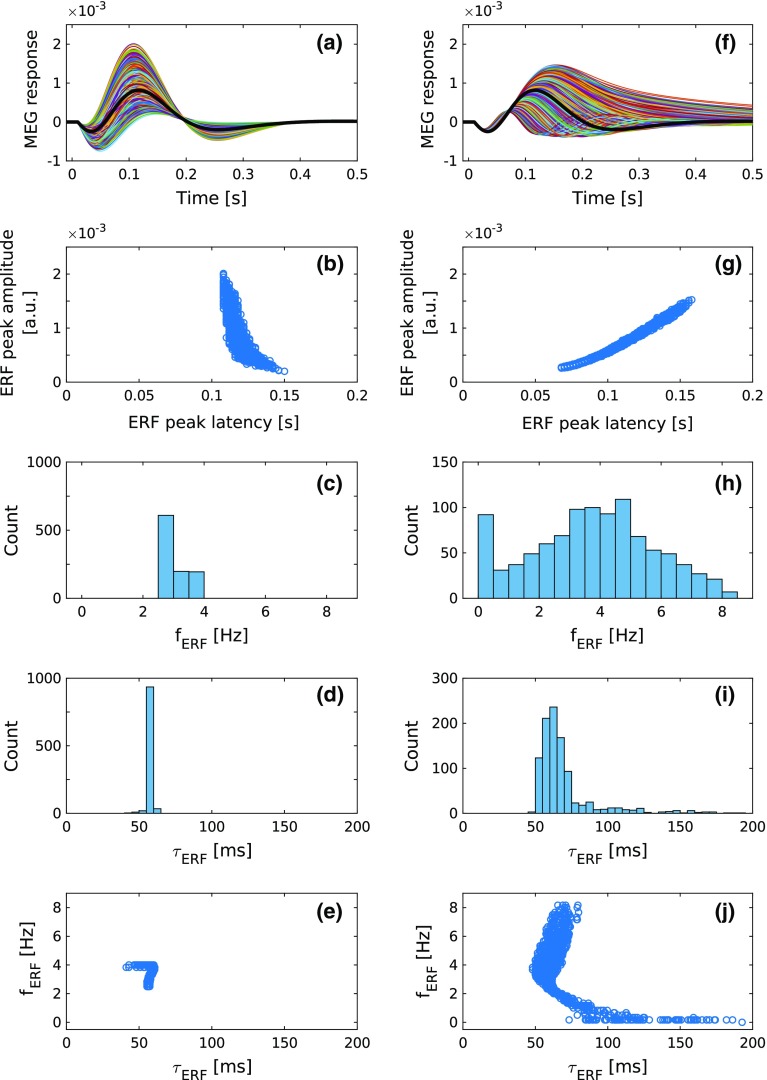
The impact of variations of the topographical parameters (left column) and dynamical parameters (right column) on the ERF. **a** The effect of topographical factors was examined by randomizing the $K_i$-matrices while keeping the parameters *P* fixed. A total of 1000 ERF waveforms were generated such that for each simulation the default value of each element of the $K_i$-matrices was multiplied by a random number from a distribution in the [0.5, 2] range. The resulting waveforms are similar in shape. **b** While the N1m-peak amplitudes of these waveforms have a wide distribution, the corresponding peak latencies inhabit a narrow range, and there is only a weak dependency between the two. **c** The spectral analysis by means of FFT reveals a narrow distribution around a frequency $f_{{\mathrm{ERF}}}$ of about 3 Hz. **d** The time constants $\tau _{{\mathrm{ERF}}}$ which describe the attenuation of the waveforms shown in **a** are narrowly distributed around 60 ms. **e** The tight distributions of $f_{{\mathrm{ERF}}}$ and $\tau _{{\mathrm{ERF}}}$ result in a rather focal distribution in the $f_{{\mathrm{ERF}}}$–$\tau _{{\mathrm{ERF}}}$ plane. **f** In studying the effects of the dynamical parameters, *P* was randomized while the $K_i$-parameters were kept constant. For each simulation, each default-valued element of the scalar matrices $\widetilde{W}_{\mathrm{ie,d}}$, $\widetilde{W}_{\mathrm{ei}}$, and $\widetilde{W}_{\mathrm{ii}}$ was multiplied by a random number from a distribution in the [0.5, 2] range. Further, for each simulation, we generated a new stochastic version of $W_{{\mathrm{AC}}}$. The resulting waveforms vary greatly in their morphology. **g** There is a strong correlation between the N1m-peak amplitude and peak latency, and both measures vary over a wide range. **h** The FFT analysis reveals a broad distribution of $f_{{\mathrm{ERF}}}$ around 4 Hz. **i** Also, $\tau _{{\mathrm{ERF}}}$ is broadly distributed around 60 Hz. **j** The broad distributions translate into an L-shaped dependency between $f_{{\mathrm{ERF}}}$ and $\tau _{{\mathrm{ERF}}}$. Note that for the randomizations of the $K_i$-matrices as well as of the *P*-parameters, two subsets of random numbers with equiprobable distributions in the [0.5, 1]-range and in the [1, 2]-range were used. Whereas the *K*-modulation yielded solely stable solutions, $19\%$ of the solutions of the *P*-modulation were unstable; these were excluded from further analysis. The thick black lines in **a** and **f** represent the default simulated waveform shown in Figs. 6b and 8b

Across-subject variability of the event-related response is likely to reflect diversity in the topography of the cortical surface. However, it is possible that variations in the dynamics of the auditory cortex also contribute. The current AC model suggests ways to tease apart these contributing factors. Given that in *P* and $K_i$ we have separate parameter sets for the dynamical and topographical contributions to the MEG signal, the question becomes whether there are aspects of the ERF which change when *P* is modulated but not when *K* is modulated, and vice versa. To this end, we examined the ERF under two conditions. In the first condition, the dynamical parameters *P* were kept constant and the topographical parameters embedded in the three *K*-matrices were varied. For this, the elements of the $K_{{\mathrm{1}}}$ matrix were grouped into a $15\times 15$ field matrix as depicted in Fig. 5a, and then each of the 88 nonzero elements of this field matrix were randomized separately by multiplying the default value (Table 1) with a random number from a distribution in the [0.5, 2] range. Similarly, the 13 nonzero elements of $K_{{\mathrm{2}}}$ and $K_{{\mathrm{3}}}$ were randomized separately using a random number from the same distribution. Figure 10a shows waveforms for 1000 such randomizations. In the second condition, the elements of the connection matrices found in *P* were randomized while the other parameters were left unchanged. For each simulation, each element of the diagonal matrices $W_{{\mathrm{ie,d}}}$, $W_{{\mathrm{ei}}}$, and $W_{{\mathrm{ii}}}$ was generated separately by multiplying the default value with a random number in the [0.5, 2] range. Also, for each simulation, we generated a new stochastic version of $W_{{\mathrm{AC}}}$ (see Appendix A1). Unstable solutions (see flowchart in Fig. 2) were excluded from further analysis. Figure 10f depicts 1000 waveforms produced this way, each one representing a stable solution.

**Fig. 11:**
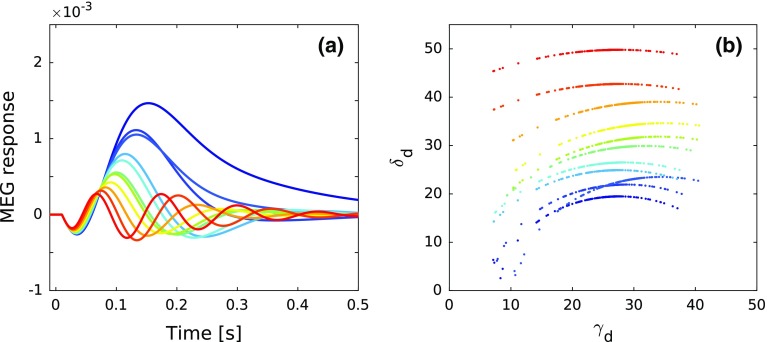
Linking ERF waveforms to normal mode parameters. **a** A subset of 11 ERF waveforms from Fig. 10f are shown. These cover the full range of $f_{{\mathrm{ERF}}}$ shown in Fig. 10h. **b** The decay constants of the underlying normal modes are plotted against the corresponding damping frequencies. Waveforms with low $f_{{\mathrm{ERF}}}$ (blue) have small $\delta _{\mathrm{d}}$ values and those with high $f_{{\mathrm{ERF}}}$ (red) have large $\delta _{\mathrm{d}}$. As $f_{{\mathrm{ERF}}}$ decreases, the dependence of $\delta _{\mathrm{d}}$ on $\gamma _{\mathrm{d}}$ increases

The randomization of the topographical parameters $K_i$ lead to a scaling of the ERF, while its overall morphology was maintained (Fig. 10a). Similar scaling effects are visible in Fig. 7 where the contribution of the $K_{{\mathrm{1}}}$ feedforward and feedback connections to the ERF are studied independently in a systematic way. In contrast, randomizing the dynamical parameters *P* resulted in a much larger diversity of the ERF waveform (Fig. 10f). To quantify these effects, we plotted the N1m-peak amplitude of the simulated waveforms against the corresponding peak latency in Fig. 10b, g. Except for the waveforms with the smallest peak amplitudes $<0.5 \times 10^{-3}$, the N1m-peak latencies of the waveforms obtained from the randomization of the topographical parameters are nearly independent of the peak amplitude and cover a narrow range between about 110 ms and 130 ms (Fig. 10b). In contrast, when the dynamical parameters are randomized, the N1m-peak latencies span a much wider range from approximately 70 ms to 160 ms (Fig. 10g). Further, we observe a strong correlation between peak amplitude and peak latency.

The diversity between the waveform morphology can further be expressed in terms of Fourier frequency $f_{{\mathrm{ERF}}}$ and decay time $\tau _{{\mathrm{ERF}}}$ of the waveforms. The Fourier frequencies $f_{{\mathrm{ERF}}}$ shown in Fig. 10c, h were obtained through a standard fast Fourier transform (FFT) and represent the dominant frequency of the FFT analysis. As expected from Fig. 10a, variations in the topographical parameters resulted in a narrow distribution of $f_{{\mathrm{ERF}}}$ around 3 Hz. By comparison, when the dynamical parameters were varied, the distribution was much broader and peaked at 4 Hz. Note that the increase in the distribution at lower values of $f_{{\mathrm{ERF}}}$ is due to those broad MEG waveforms in Fig. 10f that do not reach baseline level even by $t = 500$ ms. The time constant $\tau _{{\mathrm{ERF}}}$ describes the temporal decay of each waveform, and it was determined by first calculating the envelope of the ERF through the application of the Hilbert transform to the data. In a second step, an exponential decay function was fitted to the transformed data in a time interval ranging from the peak value of the envelope, at around 100 ms, to 600 ms where the MEG signal had sunk back to its baseline level. For both topographical and dynamical variations, the distribution of $\tau _{{\mathrm{ERF}}}$ was centered in the 60–70 ms range. However, the distribution of $\tau _{{\mathrm{ERF}}}$ was much broader for the dynamical variations than the topographical ones, as shown in Fig. 10d, i.

Further differences between the effects of topographical and dynamical variations become evident when $f_{{\mathrm{ERF}}}$ is plotted against $\tau _{{\mathrm{ERF}}}$. In the case of topographical variations, the tight distributions of these morphological descriptors depicted in Fig. 10c, d translate into a rather focal distribution in the $f_{{\mathrm{ERF}}}$–$\tau _{{\mathrm{ERF}}}$ plane, as shown in Fig. 10e. Interestingly, the broader distributions of $f_{{\mathrm{ERF}}}$ and $\tau _{{\mathrm{ERF}}}$ associated with dynamical variations did not translate into an even or random distribution in the $f_{{\mathrm{ERF}}}$–$\tau _{{\mathrm{ERF}}}$ plane. Instead, Fourier frequency and temporal decay showed a dependency on each other, with the distribution forming a distinct L shape, as is evident in Fig. 10j. Thus, there were two regions in the distribution: in the narrow range of $\tau _{{\mathrm{ERF}}} = ({60} \pm 10)$ ms, the corresponding $f_{{\mathrm{ERF}}}$ had a wide distribution extending from 2 to 8 Hz. Conversely, when $f_{{\mathrm{ERF}}}$ was below 1 Hz, $\tau _{{\mathrm{ERF}}}$ was distributed over a 70–200 ms range. Thus, there were no instances of fast temporal decay of the ERF waveform coupled with a high Fourier frequency.

Finally, we linked the variations in the ERF waveforms back to the parameters which characterize the normal modes. Figure 11a shows a subset of the ERFs shown in Fig. 10f covering a broad range of $f_{{\mathrm{ERF}}}$. For each ERF, we plotted the damping frequency $\delta _{\mathrm{d}}$ against the decay constant $\gamma _{\mathrm{d}}$ of the 240 underlying normal modes in Fig. 11b. While there was little variation of $\gamma _{\mathrm{d}}$ across the different ERFs, $\delta _{\mathrm{d}}$ varied over a wide range not only in its absolute values but also in the dependence on $\gamma _{\mathrm{d}}$. For ERFs with low $f_{{\mathrm{ERF}}}$ (blue curves in Fig. 11a), $\delta _{\mathrm{d}}$ has small values and shows a strong dependence on $\gamma _{\mathrm{d}}$. As $f_{{\mathrm{ERF}}}$ increases, so does $\delta _{\mathrm{d}}$, and the dependence of $\delta _{\mathrm{d}}$ on $\gamma _{\mathrm{d}}$ becomes weaker.

In summary, these results predict that variations in the ERF waveform are specific to the type of parameter that is being varied. Thus, variations in dynamical parameters lead to a much broader selection of waveforms than do changes in topographic parameters. These results, depicted in Fig. 10, serve as predictions for testing in ERF measurements. We have confirmed these findings using multiple default models with realistic-looking N1m–P2m responses.

## Discussion

Here, we presented a mechanistic explanation of long-latency auditory ERFs by developing analytical solutions for an already existing nonlinear model of AC signal processing. The model is based on the idiosyncratic architecture of AC in which information flows in a distinctly serial manner along multiple parallel streams within a core–belt–parabelt structure. We derived analytical solutions of the coupled differential equations for the state variables of the excitatory and inhibitory cell populations by assuming that the response to the synaptic input is linear in a wide range of spiking rates, and by using symmetric connections between the cell populations. The result is a description of the system dynamics in terms of normal modes, that is, decoupled damped harmonic oscillators. The ERF response reflects these dynamics but it is modulated by a set of non-dynamical factors comprising the topography of the primary currents and the effects of the type of connection contributing to the primary current. We showed that the ERF response originates from a mixture of normal modes, and that these directly depend on the anatomical structure as expressed in the connection matrices. In our account, each peak and trough of the ERF is not due to dedicated response generators but, rather, arises out of the network properties of the entire AC. The model generates predictions for testing whether the large inter-subject variability of ERFs is due merely to subject-specific cortical topographies or whether it also reflects subject-specific cortical dynamics.

### The link between anatomy, dynamics, and ERFs

The current work accounts for auditory ERFs by decomposing them into a set of normal modes. Each normal mode is a solution to the equations for a driven damped harmonic oscillator [Eqs. (13) and (14)], and falls into one of three types: overdamped, critically damped, or underdamped. Further, a normal mode is defined by its amplitude [Eq. (15) or (16)] as well as by two physical terms, the decay constant $\gamma _{\mathrm{d}}$ and the damping frequency $\delta _{\mathrm{d}}$ [Eq. (17)]. These parameters are, in turn, functions of the set of dynamical parameters we denote by *P*, which includes all the connection matrices. With each normal mode depending directly on the entire set of connection patterns and connection strengths of the system, the decomposition of the ERF into normal modes anchors the ERF waveform directly to the anatomical structure of AC. Thus, modifying the anatomical structure can change subtle aspects of the ERF, such as the amplitudes and latencies of individual peaks and troughs. However, anatomical structure also determines what the mixture of the normal modes are in terms of their type, and it is therefore reflected in the gross aspects of the ERF, that is, whether certain peaks and troughs appear at all.

We also see how the activity of each individual column depends not just on the synaptic input to the column but, rather, it directly reflects the entire anatomical structure of the AC. The connection matrix $W_{{\mathrm{AC}}}$ (Fig. 1b) plays a special role in the model. It consists of all the short- and long-range connections, including those which relay lateral inhibition, and thus encapsulates the anatomical structure of AC. Thus, for any specific pattern of connections and set of connection strenghts, $\widetilde{W}_{\mathrm{AC}}$ will have a specific set of eigenvalues and eigenvectors. For a given set of $W_{{{\mathrm{ei}}}}$, $W_{{{\mathrm{ii}}}}$ and $W_{{{\mathrm{ie,d}}}}$ matrices, the eigenvalues of $\widetilde{W}_{\mathrm{AC}}$ define the distribution of frequencies $\delta _{\mathrm{d}}$ of the normal modes [Eq. (17)]. Further, the eigenvectors regulate how the input is distributed among the normal modes [see Eqs. (45) and (46) in Appendix A2]. Using the eigenvectors gathered in the matrix $\varUpsilon$ to couple the normal modes [Eq. (18)] gives expression to the state variables $\mathbf u (t)$ and $\mathbf v (t)$. Consequently, the state variable of any single column is a representation of all the normal modes, which themselves are functions of the structure of the AC network. Introducing variations in $\widetilde{W}_{\mathrm{AC}}$ leads to changes in its eigenvalues and eigenvectors and, thus, in the dynamics of the system both on the single-column level and in terms of the ERF. We generated multiple $\widetilde{W}_{\mathrm{AC}}$ matrices, and observed that $\delta _{\mathrm{d}}$ depended strongly on the connection strengths while the distribution of $\gamma _{\mathrm{d}}$ was little affected (Fig. 11). ERFs with a single peak were produced by systems with a relatively wide distribution of low-valued $\delta _{\mathrm{d}}$ which showed a strong dependence on $\gamma _{\mathrm{d}}$. Multi-peaked ERFs were generated by systems with high-valued $\delta _{\mathrm{d}}$ packed into a narrow range, with little dependence on $\gamma _{\mathrm{d}}$.

The normal modes are dynamic units which cannot be localized to any particular single location in AC. Each one can be thought of as being spread over the whole AC in a unique fashion, contributing to the activity of the cortical columns with varying strengths and polarities. This spread is accessible in the analytical approach, allowing one to map the mean contribution that each normal mode makes to each cortical field (Fig. 4). The resulting anatomical maps of the normal modes tended to show that high damping frequencies were associated with increased spatial structure. Though not shown explicitly here, one upshot of this is that the early part of the ERF is generated by normal modes with large variations across cortical fields, resulting in lower field-to-field correlations in their activity. In contrast, the late part of the ERF is dominated by normal modes with a uniform effect over the fields, resulting in higher inter-field correlations. This view opens up the possibility to consider how the activity of different fields and columns are coupled to each other via the normal modes in dynamic connectivity maps. The full implications of these observations and resulting predictions will be returned to elsewhere.

The MEG signal arises out of the cortical primary currents, which are driven by the system dynamics, and this signal is modulated by topographical factors influencing the orientation of the current. One of these factors is whether the connection driving the primary current represents feedforward or feedback input to the cortical column (Ahlfors et al. 2015). As part of the dynamical parameters *P*, our model included both feedforward and feedback AC connections in the connection matrix $W_{{\mathrm{AC}}}$ (and the corresponding $\widetilde{W}_{\mathrm{AC}}$). The differential contributions of these two kinds of connections to the MEG signal was approximated through the use of the topographical $K_{{\mathrm{1}}}$ matrix. By systematically varying the size of these contributions (while keeping the dynamics fixed), we found that the P1m reflects primarily feedforward activation and that feedback activations drive the N1m and P2m (Fig. 7). Due to the fixed dynamics, this investigation did not address how the feedback connections contribute to the dynamics of AC. A natural way to address this question would be to modify the actual feedback connections in $W_{{\mathrm{AC}}}$ (and in $\widetilde{W}_{\mathrm{AC}}$ respectively). However, our analytical approach does not allow this because of the requirement of symmetric connection matrices, and therefore numerical simulations would be required. We note that the role of feedback connections in neural processing is, in general, an open question, with earlier accounts labeling them weak and modulatory (Crick and Koch 1998; Sherman and Guillery 2011) and the predictive-coding framework requiring them generally to be functionally inhibitory (Bastos et al. 2012). In our model, feedback connections were symmetric with feedforward connections (Felleman and Essen 1991), as well as excitatory and of the driving kind (Covic and Sherman 2011). Leaving this to be addressed elsewhere, we suspect that feedback connections contribute to a larger $\tau _{{\mathrm{ERF}}}$ and/or a lower $f_{{\mathrm{ERF}}}$. That is, they might act as a memory mechanism by keeping the signal circulating in the AC for longer. This, in turn, might be beneficial for enhancing the signal-to-noise ratio in auditory processing, or for allowing the build-up of synaptic depression, which might be instrumental for representing the temporal structure of sound (May and Tiitinen 2013; May et al. 2015; Westö et al. 2016).

### A novel approach for ERF generation

#### State of the art: ECD source localization and its variations

In MEG research, the ERF waveform is usually treated as a linear combination of the activity of spatially distributed sources in the brain, and the task becomes one of localizing and modeling the activity of each source. Accurate localization of MEG sources, however, suffers from the ill-posed inverse problem. As solutions to this problem, numerous approaches have been developed, including discrete and distributed source models (Mosher et al. 1992; Scherg 1990; Scherg and Berg 1996), along with several variants of beamformers, a spatial filtering technique often applied in the analysis of brain oscillations (see, for example, Darvas et al. 2004; Hillebrand and Barnes 2005; Wendel et al. 2009).

Discrete and distributed source models use time-varying ECDs as the simplest physiologically meaningful source model. The mathematical concept of the ECD is a point-like source, and it is an abstraction which is justified in those cases where the spatial extension of the activated brain region is small compared to its distance to the MEG sensors. For example, it is common practice in experiments with simple auditory stimuli to use a single ECD per hemisphere to explain the measured magnetic field distribution describing the N1m waveform as the result of a best match between forward and inverse solution. The multi-dipole model is often used when the brain activation can be described by a small number of stationary focal sources, which is commonly the case in simple sensory experiments. To determine an adequate number of sources, a conservative and rather subjective approach is to gradually increase the number of sources on condition that for each source a distinct contribution to the measured magnetic field pattern is verifiable, i.e. that the sources do not model noise. More exacting approaches use advanced classification algorithms, such as the “recursively applied and projected multiple signal classification” method (RAP-MUSIC; Mosher and Leahy 1999). In case of spatially extended brain activation, the concept of discrete sources is often replaced by distributed source models estimating simultaneously strengths and directions of dipoles located on a grid of hundreds or even thousands of brain locations (see, for example, Dale and Sereno 1993; Hämäläinen and Ilmoniemi 1994; Dale et al. 2000; Pascual-Marqui 2002).

Taken together, the ECD as source model is a simplification which makes the source localization problem mathematically tractable. However, there are numerous arbitrary choices that the researcher has to make. Notably, these include an a priori assumption on the number of sources in discrete source analysis, and constraints such as regularization parameters in distributed source models. Therefore, source localization carries with it unavoidable ambiguities: Has the correct number of underlying neural sources been assumed? Have these been reliably and correctly separated from each other, in particular when two or more sources are close to each other in space and in time (Lütkenhöner and Steinsträter 1998)? Thus, precise source localization based on trial-averaged ERFs is non-trivial not only due to the inverse problem per se, but also due to the unknown number of sources and their separability.

#### An alternative view on ERF generators

With conventional source modeling, the temptation is to understand an ERF generator in terms of a spatially and temporally constrained local process giving rise to a “component” of the ERF (Näätänen and Picton 1987; Näätänen 1992)—in effect equating sources (i.e., the primary currents) with generators (i.e., the neural tissue with the processes generating the primary currents). Thus, for example, the P1m generators are those cortical areas which are active during the peak of the P1m, and the objective of source modeling is to localize these generators. Conversely, each cortical area might be considered a generator of a component, and the challenge for source localization is to separate these generators out from each other so that the component structure of the event-related response can be identified. It follows that if the sources identified for the P1m are found to be different than those active during the N1m peak, the conclusion can be drawn that the P1m and the N1m have at least partially different (though possibly overlapping) generators. In this vein, cortical activation can be seen as signal propagation as successive generators become active, as is evident in source modeling assuming a single ECD (Lütkenhöner and Steinsträter 1998), multiple dipoles (Inui et al. 2006) and distributed sources (Yvert et al. 2005).

Our model opens up an alternative view on ERF generation. Rather than considering the ERF to be the linear sum of multiple spatially discrete sources, it becomes a combination of multiple normal modes. Here, the normal modes themselves and the way they are coupled are determined by the anatomical structure of AC and by other, dynamical parameters. This approach still approximates the ERF-generating system as a set of discrete sources—cortical columns—but it lays emphasis on the way these are connected to each other and to the dynamics of this connected system. In this way, the system can be described on three distinct levels: that of physiological and anatomical quantities, that of the normal modes, and that of the primary currents which are determined by the normal modes.

What, then, is an ERF generator in this normal mode view? In our simulations, the AC has well-defined sources of activity—those columns activated by the stimulus—and each field and area has a well-defined contribution to the ERF. Also, there is a serial progression of activation along the core–belt–parabelt axis (Figs. 3, 8), which fits in with experimental observations (Inui et al. 2006; Yvert et al. 2005; Guéguin et al. 2007). As such, these results add nothing to the conventional view of ERF generation: in our simulations, the main generators of the N1m response are clearly the core and belt areas; with the parabelt contributing very little, it seems clear that it cannot be counted as an N1m generator. However, this view is countered by the consideration that both single-column activity and the ERF represent the combination of multiple normal modes and that each normal mode is a function of the connection patterns and strengths of the entire AC system. Thus, the local connections in the parabelt, and so the parabelt fields themselves, are an intimate part of activity generation in the core and belt. This, in turn, means that one cannot consider individual columns, fields, or areas as separable ERF generators. The parabelt is just as much an N1m generator as the core and, similarly, the core is just as much a P2m generator as the parabelt.

The above principle of ERF generation is demonstrated in Fig. 9 which shows the effects of modifying the local connections within the parabelt fields. These modifications lead to modest changes in the parabelt response itself. Importantly, they entail significant changes in the activity of the core and belt. This finding is all the more intriguing since the contributions of the two parabelt fields to the overall ERF are significantly smaller than those of the core and belt (see Fig. 8b). Thus, while the parabelt does not function as a source of the N1m, it is clearly an important part of the N1m generator.

On a more fundamental level, there is an ongoing debate about the generation of event-related responses (de Munck and Bijma 2010; Sauseng et al. 2007; Telenczuk et al. 2010; Turi et al. 2012; Yeung et al. 2004). In the classical signal-plus-noise (SPN) model, the trial-averaged MEG response is treated as the superposition of a stationary stimulus-evoked signal and zero-mean Gaussian noise. In this view, an ERF is a time-locked phasic burst which is uncorrelated with the ongoing rhythmic activity (Arieli et al. 1996; Dawson 1954; Mäkinen et al. 2005; Mazaheri and Jensen 2006; Shah et al. 2004). The phase-reset model proposed by Sayers et al. (1974) (see also Makeig et al. 2002; Hanslmayr et al. 2007) provides an opposing view according to which stimulus-evoked responses are generated by partial stimulus-induced phase synchronization of the rhythmic background activity. The most recent model for ERF generation is the baseline-shift model introduced by Nikulin et al. (2007, 2010). This model is based on the asymmetric modulation of the amplitude of spontaneous alpha-band oscillations, although de Munck and Bijma (2010) argue that it could be viewed as a special case of the SPN model. The current results of our work do not as such contribute to this debate because we did not include oscillatory background activity in the simulations. However, while beyond the scope of the current study, such oscillatory activity would be easy enough to include in the model. The oscillator nature of the model implies that feeding the model with noise should already be sufficient to generate ongoing oscillations. Alternatively, the analytical solutions themselves show that the resting state of the model would be a limit cycle if the decay constant $\gamma _{\mathrm{d}}$ for one or more normal modes in Eqs. (13) and (14) is zero. A stimulus acting as an outside push to the individual harmonic oscillators represented by the normal modes could have a multitude of effects, depending on the amplitude of the ongoing oscillations, and this could be approached analytically by considering the unit impulse response of our model. Further, as our model operates on multiple spatial resolutions, from the single-column to the aggregated MEG signal, it might be useful for approaching the question of whether phasic responses and ongoing oscillations are generated by the same or different neural populations (Sauseng et al. 2007).

### Subject-specificity of the event-related waveforms

Event-related responses are characterized by large between-subject variability. Although rarely an object of study, this variability is evident to any researcher using ERPs and ERFs (Luck 2014). It is also given expression in, for example, the large standard deviations of peak amplitudes and latencies in test–retest studies in which the reliability and reproducibility of event-related responses have been investigated in various subject populations (e.g., Michalewski et al. 1986; Kileny and Kripal 1987; Segalowitz and Barnes 1993; Dalebout and Robey 1997; Atcherson et al. 2006). These studies tend to show that between-subject variability is contrasted by the responses staying stable for a given subject across different measurement sessions and over long periods.

There are potentially two sources of the variability of event-related responses across subjects: the anatomical topography of the cortical surface and the dynamics of AC. Subject-specific topography is well-documented, with human subjects having large differences in the pattern and number of convolutions on the supratemporal plane (Yvert et al. 2005; Moerel et al. 2014). However, the question of subject-specific dynamics of AC has, to our knowledge, not been approached before. The current AC model allowed us to address this issue in simulations via separable sets of parameters: the *P*-parameters governing the system dynamics, and the $K_i$-matrices capturing the topographical properties influencing the MEG signal. We introduced random variations to these parameter sets and found that variations of the dynamical parameters have a much stronger effect on the waveform than variations of the topographical parameters. Specifically, *K*-randomizations mainly affect the N1m-peak amplitude while keeping intact the morphology of the waveform of the entire ERF (Fig. 10a). In contrast, *P*-randomizations result in a much larger variety of waveform morphologies (Fig. 10f), with N1m-peak amplitudes and latencies varying in a considerably larger range (Fig. 10g). Consequently, changes of dynamics parameters entail a much broader FFT frequency spectrum, and, likewise, a broader distribution of the time constant $\tau _{{\mathrm{ERF}}}$ (Fig. 10h, i).

These results provide predictions for straightforward testing in populations of subjects: Characterizing single-subject ERFs in terms of frequency spectrum and $\tau _{{\mathrm{ERF}}}$, how are these estimates distributed over the population? If the distribution is narrowly focused, this would indicate that subjects have similar AC dynamics and that the subject-specificity of the ERF is due to topographical variations only. In contrast, subject-specific dynamics would be indicated by a wider distribution of frequency spectrum and $\tau _{{\mathrm{ERF}}}$, especially if this distribution is structured as in Fig. 10e. Previous research has pointed to a large variation in the N1/N1m peak latency (e.g., Michalewski et al. 1986; Kileny and Kripal 1987; Segalowitz and Barnes 1993; Dalebout and Robey 1997; Atcherson et al. 2006), and this would fit with the results of the current simulations utilizing dynamical parameter randomizations (Fig. 10g). Thus, we would expect to see evidence supporting the presence of subject-specific AC dynamics.

While our results suggest that the separation of dynamical and topographical effects might be possible on the population level, is there hope for such a separation when looking at single-subject data? This is a challenge since (random) combinations of *P*- and *K*-parameters lead to a wealth of waveforms. One way forward might be through systematic investigations of how waveform properties depend on parameters. This might enable us to identify major causal relationships between dynamical and topographical parameters on the one hand and the resulting waveforms on the other. One compelling example is our finding that the feedforward projections of the $K_{{\mathrm{1}}}$-matrix are crucial for the generation of the P1m response.

The current model is based on the architecture of the monkey AC (Kaas and Hackett 2000; Hackett et al. 2014), as a comparable map of the organization of human AC is still missing (Nourski et al. 2014; Leaver and Rauschecker 2016). Might our approach offer a method for fitting anatomical organization of the AC to the ERF in humans? This would present an inverse problem quite different from the source localization one. Instead of using ECDs, the solution would be expressed in terms of normal modes and the underlying architecture coupled with the subject-specific cortical surface. Even in the presence of subject-specific ERF waveforms, this approach might become possible with the constraint that the coarse architecture in terms of cortical fields and their interconnections is a shared feature across human subjects. The first steps in this investigation will require computational studies on the effect of architecture on the ERF waveforms. Specifically, can species-specific event-related responses be explained by a species-specific constellation of fields in auditory cortex? Further, might the distributions of ERF descriptors such as those presented in Fig. 10 be used to decode anatomical structure on the population level?

## Figures and Tables

**Fig. 12:**
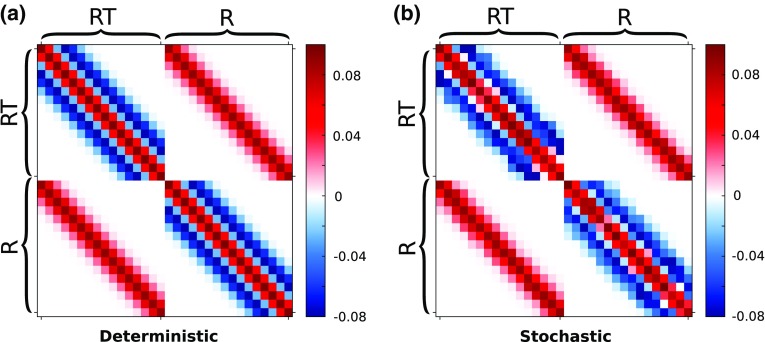
The spread of between-field and within-field connections of $W_{{\mathrm{AC}}}$ described by a Gaussian distribution *Q*(*x*). The core fields **RT** and **R** are used for illustration. **a** In the deterministic case, the parameter *s* which describes the level of stochasticity, was set to zero. The resulting connection pattern between column *i* and *j* depends solely on the distance between $|i-j|$. **b** In this example for a stochastic case, the parameter *s* was set to 0.2 and 0.4 for between- and within-field connections, respectively (see Table 2). Hence, the weight value assigned to the connection between column *i* and *j* is modified by a random value. Note that the scales to the right of both matrices indicate the sign (polarity) of the weights which is positive for excitatory (red) or negative for inhibitory (blue) connections

## Appendix

### A1: Connection spread in $W_{{\mathrm{AC}}}$ described by means of Gaussian distributions

All inter-column connections are contained in $W_{{\mathrm{AC}}}$ as displayed in Fig. 1b. Here, $W_{{\mathrm{AC}}}$ refers to the connection matrix that contains all lateral and long-range connections (see Appendix A2). A cortical column at position *i* in the field is connected to itself and to neighboring columns *j*. Similarly, when making connections to another field, the column targets its tonotopic counterpart *i* as well as columns *j* in the neighborhood of this counterpart. In both cases, the spread of connections is described by a Gaussian distribution $Q(x) = r \exp [-(x + \mu + s\ \mathcal {N}(0,1))^2/2\sigma ^2]$ where *x* is the tonotopic distance $|i-j|$, *r* is a scaling parameter, the variance $\sigma ^2$ determines the width of the spread, $\mu$ is an offset parameter, *s* determines the level of stochasticity of the connections, and $\mathcal {N}(0,1)$ is the standard normal distribution. The default parameter values used for *Q*(*x*) are listed in Table 2. As an example, Fig. 12a, b demonstrates the spread in the connections between and within the two core fields RT and R both in the deterministic ($s=0$) and stochastic ($s>0$) case, respectively.

**Table 2:** Default parameter values of the amplitude *r*, constant $\mu$, variance $\sigma ^2$, and stochasticity *s* of the Gaussian distributions *Q*(*x*) for computing the between-field and within-field weights of $W_{{\mathrm{AC}}}$

	*r*	$\mu$	$\sigma ^2$	*s*
Between-field excitatory	0.09	0	1.5	0.2
Within-field excitatory	0.105	0	2.0	0.4
Within-field inhibitory	0.09	3.0, $-$ 3.0	1.5	0.4

*Between-field connections *These are all excitatory and described by a single Gaussian with $r=0.09$, $\mu = 0$, $\sigma ^2 = 1.5$. They are shown in the lower-left and upper-right quadrants of Figs. 12a ($s = 0$) and 12b ($s = 0.2$).

*Within-field connections* These are described by a sum of Gaussians so that excitatory connections for nearest neighbors give way to lateral inhibitory connections at larger distances. Excitatory weights are computed using $Q_{{\mathrm{exc}}}(x)$ with $r=0.105$, $\mu = 0$, $\sigma ^2 = 2.0$. For lateral inhibition, we have two Gaussians $Q_{{\mathrm{inh,1}}}(x)$ and $Q_{{\mathrm{inh,2}}}(x)$, both with $r=0.09$, $\sigma ^2 = 1.5$, but with $\mu = -3.0$ and $\mu = 3.0$, respectively. The total weights are $Q_{{\mathrm{intra}}}(x) = Q_{{\mathrm{exc}}}(x) - [Q_{{\mathrm{inh,1}}}(x) + Q_{{\mathrm{inh,2}}}(x)]$. These are shown in the upper-left and lower-right quadrants in the matrices in Figs. 12a ($s = 0$) and 12b ($s = 0.4$).

### A2: Derivation of analytical solutions

As introduced in Sect. 2, the dynamics of the system are determined by $2 \times N$ coupled nonlinear differential equations [see Eqs. (1) and (2)]:

20 $$\begin{aligned}&\begin{aligned} \tau _{\mathrm{m}}\dot{\mathbf{u }}(t)&+\mathbf u (t) - A(t)\circ W_{\mathrm{ee}} \cdot g[\mathbf u (t)] \\&\quad +\, W_{\mathrm{ei}}\cdot g[\mathbf v (t)] = \mathbf I _{\mathrm{aff,e}}(t) \end{aligned} \end{aligned}$$

21 $$\begin{aligned}&\begin{aligned} \tau _{\mathrm{m}}\dot{\mathbf{v }} (t)&+ \mathbf v (t) - W_{\mathrm{ie}} \cdot g[\mathbf u (t)] \\&\quad +\, W_{\mathrm{ii}}\cdot g[\mathbf v (t)] = \mathbf {I}_{\mathrm{aff,i}}(t). \end{aligned} \end{aligned}$$

Ignoring the synaptic plasticity term *A*(*t*) and inserting the linear spiking-rate function $g(x) = \alpha x$ into Eqs. (20) and (21) results in the linearized state equations

22 $$\begin{aligned}&\begin{aligned}&\tau _{\mathrm{m}}\dot{\mathbf{u }}(t) + \mathbf u (t) - W_{\mathrm{ee}}\cdot [\alpha \mathbf u (t)] \\&\quad +\, W_{\mathrm{ei}}\cdot [\alpha \mathbf v (t)] = \mathbf I _{\mathrm{aff,e}}(t) \end{aligned} \end{aligned}$$

23 $$\begin{aligned}&\begin{aligned}&\tau _{\mathrm{m}}\dot{\mathbf{v }}(t) + \mathbf v (t) - W_{\mathrm{ie}}\cdot [\alpha \mathbf u (t)] \\&\quad +\, W_{\mathrm{ii}}\cdot [\alpha \mathbf v (t)] = \mathbf I _{\mathrm{aff,i}}(t). \end{aligned} \end{aligned}$$

Dividing Eqs. (22) and (23) by $\tau _{\mathrm{m}}$ leads to

24 $$\begin{aligned}&\dot{\mathbf{u }}(t) - \widetilde{W}_{\mathrm{ee}}{} \mathbf u (t) + \widetilde{W}_{\mathrm{ei}}{} \mathbf v (t) = \mathbf I _{\mathrm{e}}(t) \end{aligned}$$

25 $$\begin{aligned}&\dot{\mathbf{v }}(t) - \widetilde{W}_{\mathrm{ie}}{} \mathbf u (t) + \widetilde{W}_{\mathrm{ii}}{} \mathbf v (t) = \mathbf I _{\mathrm{i}}(t) , \end{aligned}$$

with

26 $$\begin{aligned} \left\{ \begin{array}{@{}ll@{}} \frac{\alpha W_{\mathrm{ee}} - I}{ \tau _{\mathrm{m}}}&{}\longrightarrow \widetilde{W}_{\mathrm{ee}} \\ \frac{\alpha W_{\mathrm{ei}}}{\tau _{\mathrm{m}}}&{}\longrightarrow \widetilde{W}_{\mathrm{ei}} \\ \frac{\alpha W_{\mathrm{ie}}}{\tau _{\mathrm{m}}}&{}\longrightarrow \widetilde{W}_{\mathrm{ie}} \\ \frac{\alpha W_{\mathrm{ii}} + I}{\tau _{\mathrm{m}}}&{}\longrightarrow \widetilde{W}_{\mathrm{ii}} \\ \frac{\mathbf{I }_{{\mathrm{aff,e}}}(t)}{\tau _{\mathrm{m}}}&{}\longrightarrow \mathbf I _{\mathrm{e}}(t) \\ \frac{\mathbf{I }_{{\mathrm{aff,i}}}(t)}{\tau _{\mathrm{m}}}&{}\longrightarrow \mathbf I _{\mathrm{i}}(t). \end{array}\right. \end{aligned}$$

The matrix terms with the capping tilde represent the connection matrices of the linearized canonical form. To obtain analytical solutions of this system of first-order differential equations, we transform it into a system of second-order differential equations:

27 $$\begin{aligned}&\ddot{\mathbf{u }}(t)+2\varGamma _u\dot{\mathbf{u }}(t)+\varOmega ^2_{0,u}{} \mathbf u (t)=\mathbf q (t) \end{aligned}$$

28 $$\begin{aligned}&\ddot{\mathbf{v }}(t)+2\varGamma _v\dot{\mathbf{v }}(t)+\varOmega ^2_{0,v}{} \mathbf v (t)=\mathbf j (t), \end{aligned}$$

with the definition of the coefficients given in Eqs. (5)–(10). Analytical solutions for $\mathbf u (t)$ and $\mathbf v (t)$ of Eqs. (27) and (28) can be found if $\varGamma _u$ and $\varOmega ^2_{0,u}$ as well as $\varGamma _v$ and $\varOmega ^2_{0,v}$ are simultaneously diagonalizable.

The matrices $\varGamma _u$ and $\varOmega ^2_{0,u}$ as well as $\varGamma _v$ and $\varOmega ^2_{0,v}$, are simultaneously diagonalizable if there exist single invertible matrices $\varUpsilon _u$ and $\varUpsilon _v$ such that

29 $$\begin{aligned} \varGamma _{u_{\mathrm{d}}}= & {} \varUpsilon ^{-1}_u\varGamma _u \varUpsilon _u \Longleftrightarrow \varUpsilon _u\varGamma _{u_{\mathrm{d}}}\varUpsilon ^{-1}_u=\varGamma _u \end{aligned}$$

30 $$\begin{aligned} \varOmega ^2_{0,u_{\mathrm{d}}}= & {} \varUpsilon ^{-1}_u\varOmega ^2_{0,u} \varUpsilon _u \Longleftrightarrow \varUpsilon _u\varOmega ^2_{0,u_{\mathrm{d}}} \varUpsilon ^{-1}_u=\varOmega ^2_{0,u} \end{aligned}$$

31 $$\begin{aligned} \varGamma _{v_{\mathrm{d}}}= & {} \varUpsilon ^{-1}_v\varGamma _v \varUpsilon _v \Longleftrightarrow \varUpsilon _v\varGamma _{v_{\mathrm{d}}} \varUpsilon ^{-1}_v=\varGamma _v \end{aligned}$$

32 $$\begin{aligned} \varOmega ^2_{0,v_{\mathrm{d}}}= & {} \varUpsilon ^{-1}_v\varOmega ^2_{0,v} \varUpsilon _v \Longleftrightarrow \varUpsilon _v\varOmega ^2_{0,v_{\mathrm{d}}} \varUpsilon ^{-1}_v=\varOmega ^2_{0,v}, \end{aligned}$$

where the subscript ‘d’ indicates a diagonal matrix. Note that $\varGamma _u$, $\varOmega ^2_{0,u}$, $\varGamma _v$, and $\varOmega ^2_{0,v}$ are governed by the connection matrices [see Eqs. (5)–(8)]. Inserting Eqs. (5) and (7) into Eqs. (29) and (30), and Eqs. (6) and (8) into Eqs. (31) and (32), yields

33 $$\begin{aligned} \varGamma _{u_{\mathrm{d}}}= & {} \varUpsilon ^{-1}_u\left( \frac{\widetilde{W}_{\mathrm{ei}}\widetilde{W}_{\mathrm{ii}}\widetilde{W}_{\mathrm{ei}}^{-1}-\widetilde{W}_{\mathrm{ee}}}{2}\right) \varUpsilon _u \end{aligned}$$

34 $$\begin{aligned} \varOmega ^2_{0,u_{\mathrm{d}}}= & {} \varUpsilon ^{-1}_u(\widetilde{W}_{\mathrm{ei}}\widetilde{W}_{\mathrm{ie}}-\widetilde{W}_{\mathrm{ei}}\widetilde{W}_{\mathrm{ii}}\widetilde{W}_{\mathrm{ei}}^{-1}\widetilde{W}_{\mathrm{ee}})\varUpsilon _u \end{aligned}$$

35 $$\begin{aligned} \varGamma _{v_{\mathrm{d}}}= & {} \varUpsilon ^{-1}_v\left( \frac{\widetilde{W}_{\mathrm{ii}}-\widetilde{W}_{\mathrm{ie}}\widetilde{W}_{\mathrm{ee}}\widetilde{W}_{\mathrm{ie}}^{-1}}{2}\right) \varUpsilon _v \end{aligned}$$

36 $$\begin{aligned} \varOmega ^2_{0,v_{\mathrm{d}}}= & {} \varUpsilon ^{-1}_v\left( \widetilde{W}_{\mathrm{ie}}\widetilde{W}_{\mathrm{ei}}-\widetilde{W}_{\mathrm{ie}}\widetilde{W}_{\mathrm{ee}}\widetilde{W}_{\mathrm{ie}}^{-1}\widetilde{W}_{\mathrm{ii}}\right) \varUpsilon _v. \end{aligned}$$

To make the diagonalization feasible, we simplify the terms in the brackets on the right hand sides of Eqs. (33)–(36). Note that $\widetilde{W}_{\mathrm{ei}}$ and $\widetilde{W}_{\mathrm{ii}}$ are scalar matrices and, thus, commute with any other matrix. The matrix $\widetilde{W}_{\mathrm{ie}}$ is non-diagonal. We approximate it by a scalar matrix $\widetilde{W}_{\mathrm{ie,d}}$ by discarding all its off-diagonal elements, i.e. by effectively removing lateral inhibition from $\widetilde{W}_{\mathrm{ie}}$. We return lateral inhibition to the dynamics by replacing $\widetilde{W}_{\mathrm{ee}}$ by $\widetilde{W}_{\mathrm{AC}}$ where elements of $\widetilde{W}_{\mathrm{ee}}$ representing excitatory connections are combined with elements of $\widetilde{W}_{\mathrm{ie}}$ describing lateral inhibition. With $\widetilde{W}_{\mathrm{ei}}$, $\widetilde{W}_{\mathrm{ii}}$, and $\widetilde{W}_{\mathrm{ie,d}}$ now being scalar matrices, Eqs. (33)–(36) can be further simplified to

37 $$\begin{aligned} \varGamma _{u_{\mathrm{d}}}= & {} \frac{\widetilde{W}_{\mathrm{ii}}-\varUpsilon ^{-1}_u\widetilde{W}_{\mathrm{AC}}\varUpsilon _u}{2} \end{aligned}$$

38 $$\begin{aligned} \varOmega ^2_{0,u_{\mathrm{d}}}= & {} \widetilde{W}_{\mathrm{ei}}\widetilde{W}_{\mathrm{ie,d}}-\widetilde{W}_{\mathrm{ii}}\varUpsilon ^{-1}_u\widetilde{W}_{\mathrm{AC}}\varUpsilon _u \end{aligned}$$

39 $$\begin{aligned} \varGamma _{v_{\mathrm{d}}}= & {} \frac{\widetilde{W}_{\mathrm{ii}}-\varUpsilon ^{-1}_v\widetilde{W}_{\mathrm{AC}}\varUpsilon _v}{2} \end{aligned}$$

40 $$\begin{aligned} \varOmega ^2_{0,v_{\mathrm{d}}}= & {} \widetilde{W}_{\mathrm{ei}}\widetilde{W}_{\mathrm{ie,d}}-\widetilde{W}_{\mathrm{ii}}\varUpsilon ^{-1}_v\widetilde{W}_{\mathrm{AC}}\varUpsilon _v. \end{aligned}$$

Due to the equality of Eq. (37) with Eq. (39), and of Eq. (38) with Eq. (40), the diagonalization problem can be reduced to the simultaneous diagonalization of two matrices $\varGamma$ and $\varOmega ^2_0$

41 $$\begin{aligned} \varGamma _{\mathrm{d}}= & {} \frac{\widetilde{W}_{\mathrm{ii}}-\varUpsilon ^{-1}\widetilde{W}_{\mathrm{AC}}\varUpsilon }{2} \end{aligned}$$

42 $$\begin{aligned} \varOmega ^2_{0_{\mathrm{d}}}= & {} \widetilde{W}_{\mathrm{ei}}\widetilde{W}_{\mathrm{ie,d}}-\widetilde{W}_{\mathrm{ii}}\varUpsilon ^{-1}\widetilde{W}_{\mathrm{AC}}\varUpsilon , \end{aligned}$$

which, in turn, requires the diagonalization of $\widetilde{W}_{\mathrm{AC}}$ only. Owing to its symmetry, the matrix $\widetilde{W}_{\mathrm{AC}}$ can be diagonalized since, in general, for any given real symmetric matrix *X* it holds that $\varUpsilon ^{-1}X\varUpsilon = X_{\mathrm{d}}$, where $X_{\mathrm{d}}$ is a real diagonal matrix. The columns of the matrix $\varUpsilon$ we are searching for to diagonalize $\widetilde{W}_{\mathrm{AC}}$ are the eigenvectors of $\widetilde{W}_{\mathrm{AC}}$, and the corresponding eigenvalues are the diagonal values of $\widetilde{W}_{\mathrm{AC,d}}$.

Hence, Eqs. (27) and (28) can be simplified to

43 $$\begin{aligned} \ddot{\mathbf{u }}(t)+2\varGamma \dot{\mathbf{u }}(t)+\varOmega ^2_{0}{} \mathbf u (t)=\mathbf q (t) \end{aligned}$$

44 $$\begin{aligned} \ddot{\mathbf{v }}(t)+2\varGamma \dot{\mathbf{v }}(t)+\varOmega ^2_{0}{} \mathbf v (t)=\mathbf j (t). \end{aligned}$$

By inserting $\varGamma = \varUpsilon \varGamma _{\mathrm{d}}\varUpsilon ^{-1}$ and $\varOmega ^2_{0} = \varUpsilon \varOmega ^2_{0_{\mathrm{d}}}\varUpsilon ^{-1}$ into Eqs. (43) and (44) and multiplying the resulting equations with $\varUpsilon ^{-1}$ from the left side, we obtain

45 $$\begin{aligned} \varUpsilon ^{-1} \ddot{\mathbf{u }}(t)+2 \varGamma _{\mathrm{d}} \varUpsilon ^{-1}\dot{\mathbf{u }}(t)+ \varOmega ^2_{0_{\mathrm{d}}} \varUpsilon ^{-1}{} \mathbf u (t)= \varUpsilon ^{-1} \mathbf q (t) \end{aligned}$$

46 $$\begin{aligned} \varUpsilon ^{-1} \ddot{\mathbf{v }}(t)+2 \varGamma _{\mathrm{d}} \varUpsilon ^{-1}\dot{\mathbf{v }}(t)+ \varOmega ^2_{0_{\mathrm{d}}} \varUpsilon ^{-1}{} \mathbf v (t)= \varUpsilon ^{-1} \mathbf j (t), \end{aligned}$$

where the variables in bold are $N\times 1$ vectors, $\varUpsilon ^{-1}$ is a square matrix of order *N*, and $\varGamma _{\mathrm{d}}$ and $\varOmega ^2_{0_{\mathrm{d}}}$ are diagonal matrices of order *N*. By substituting $\ddot{\mathbf{u }}_{\mathrm{d}}(t)= \varUpsilon ^{-1} \ddot{\mathbf{u }}(t)$, $\dot{\mathbf{u }}_{\mathrm{d}}(t)=\varUpsilon ^{-1}\dot{\mathbf{u }}(t)$, $\mathbf u _{\mathrm{d}}(t)=\varUpsilon ^{-1} \mathbf u (t)$ and $\mathbf q _{\mathrm{d}}(t)=\varUpsilon ^{-1} \mathbf q (t)$ in Eq. (45), and doing similar substitutions in Eq. (46), we obtain the decoupled state equations:

47 $$\begin{aligned} \ddot{\mathbf{u }}_{\mathrm{d}}(t)+2 \varGamma _{\mathrm{d}}\dot{\mathbf{u }}_{\mathrm{d}}(t)+ \varOmega ^2_{0_{\mathrm{d}}}{} \mathbf u _{\mathrm{d}}(t)= \mathbf q _{\mathrm{d}}(t) \end{aligned}$$

48 $$\begin{aligned} \ddot{\mathbf{v }}_{\mathrm{d}}(t)+2 \varGamma _{\mathrm{d}}\dot{\mathbf{v }}_{\mathrm{d}}(t)+ \varOmega ^2_{0_{\mathrm{d}}}{} \mathbf v _{\mathrm{d}}(t)= \mathbf j _{\mathrm{d}}(t). \end{aligned}$$

Since $\varGamma _{\mathrm{d}}$ and $\varOmega ^2_{0_{\mathrm{d}}}$ in Eqs. (47) and (48) are diagonal matrices, we can replace them with their vector representations. The decoupled equations for each normal mode of the two state variables are then

49 $$\begin{aligned} \ddot{u}_{\mathrm{d}}(t)+2\gamma _{\mathrm{d}}\dot{u}_{\mathrm{d}}(t)+\omega ^2_{0_{\mathrm{d}}}u_{\mathrm{d}}(t)=q_{\mathrm{d}}(t) \end{aligned}$$

50 $$\begin{aligned} \ddot{v}_{\mathrm{d}}(t)+2\gamma _{\mathrm{d}}\dot{v}_{\mathrm{d}}(t)+\omega ^2_{0_{\mathrm{d}}}v_{\mathrm{d}}(t)=j_{\mathrm{d}}(t), \end{aligned}$$

with the solutions

51 $$\begin{aligned} u_{\mathrm{d}}(t)= & {} \exp (-\gamma _{\mathrm{d}} t)[a_{{u}_{\mathrm{d}}}\sin (\delta _{\mathrm{d}} t) + b_{{u}_{\mathrm{d}}}\cos (\delta _{\mathrm{d}} t)]+c_{{u}_{\mathrm{d}}} \end{aligned}$$

52 $$\begin{aligned} v_{\mathrm{d}}(t)= & {} \exp (-\gamma _{\mathrm{d}} t)[a_{{v}_{\mathrm{d}}}\sin (\delta _{\mathrm{d}} t) + b_{{v}_{\mathrm{d}}}\cos (\delta _{\mathrm{d}} t)]+c_{{v}_{\mathrm{d}}} . \end{aligned}$$

The coefficients $a_{{u}_{\mathrm{d}}}$, $b_{{u}_{\mathrm{d}}}$ and $c_{{u}_{\mathrm{d}}}$ as well as $a_{{v}_{\mathrm{d}}}$, $b_{{v}_{\mathrm{d}}}$, and $c_{{v}_{\mathrm{d}}}$ are given in Eqs. (15) and (16). The decay constant $\gamma _{\mathrm{d}}$ and the damping frequency $\delta _{\mathrm{d}}$ for both $u_{\mathrm{d}}(t)$ and $v_{\mathrm{d}}(t)$ are defined in Eq. (17).

With the damping frequency $\delta _{\mathrm{d}}$ being either real positive or imaginary positive as outlined in Fig. 2, the underdamped, critically damped, and overdamped oscillations have the following analytical solutions (Fowles and Cassiday 2005).

*Underdamped oscillation*: For $\delta _{\mathrm{d}} \in \mathbb {R^+}$, the expressions for $u_{\mathrm{d}}(t)$ and $v_{\mathrm{d}}(t)$ in the respective Eqs. (13) and (14) each become a product of a decaying exponential term describing the damping (since $\gamma _{\mathrm{d}} > 0$) and a sinusoidal term describing the oscillation:

53 $$\begin{aligned} u_{\mathrm{d}}(t)= \exp (-\gamma _{\mathrm{d}} t)C_{{u}_{\mathrm{d}}}\sin (\delta _{\mathrm{d}} t+\phi _{{u}_{\mathrm{d}}})+c_{{u}_{\mathrm{d}}}, \end{aligned}$$

with $C_{{u}_{\mathrm{d}}} = \sqrt{a_{{u}_{\mathrm{d}}}^2 + b_{{u}_{\mathrm{d}}}^2}$ and $\phi _{{u}_{\mathrm{d}}} = {{\,\mathrm{atan2}\,}}(b_{{u}_{\mathrm{d}}} , a_{{u}_{\mathrm{d}}})$. The same formulation applies also to $v_{\mathrm{d}}(t)$ with $C_{{v}_{\mathrm{d}}}$ and $\phi _{{v}_{\mathrm{d}}}$. The system oscillates with a decaying magnitude that approaches zero.

*Critically damped oscillation*: The general solution for critical damping, i.e., for the case $\delta _{\mathrm{d}} = 0$, is given by

54 $$\begin{aligned} u_{\mathrm{d}}(t)= \exp (-\gamma _{\mathrm{d}} t)(a'_{{u}_{\mathrm{d}}}t+b_{{u}_{\mathrm{d}}})+c_{{u}_{\mathrm{d}}} \end{aligned}$$

with

55 $$\begin{aligned} \begin{aligned} a'_{{u}_{\mathrm{d}}}&= \frac{w_{\mathrm{ei}}\gamma _{\mathrm{d}}}{\omega ^2_{0_{\mathrm{d}}}} I_{\mathrm{i}}(t)+\frac{\omega ^2_{0_{\mathrm{d}}}+w_{\mathrm{ei}} w_{\mathrm{ie,d}}-w^2_{\mathrm{ii}}}{2\omega ^2_{0_{\mathrm{d}}}} I_{\mathrm{e}}(t)\\&\quad +\,\frac{w_{\mathrm{ii}}+w_{{\mathrm{AC,d}}}}{2}u_0-w_{\mathrm{ie,d}}v_0.\\ \end{aligned} \end{aligned}$$

In the critically damped case, the system approaches zero without oscillations in the fastest possible way.

*Overdamped oscillation:* For $\delta _{\mathrm{d}} \in \mathbb {I^+}$, Eq. (51) turns into

56 $$\begin{aligned} \begin{aligned} u_{\mathrm{d}}(t)&= \exp (-(\gamma _{\mathrm{d}}-|\delta _{\mathrm{d}}|)t)\left( \frac{b_{{u}_{\mathrm{d}}}+|a_{{u}_{\mathrm{d}}}|}{2}\right) \\&\quad +\,\exp (-(\gamma _{\mathrm{d}}+|\delta _{\mathrm{d}}|)t)\left( \frac{b_{{u}_{\mathrm{d}}}-|a_{{u}_{\mathrm{d}}}|}{2}\right) +c_{{u}_{\mathrm{d}}}. \end{aligned} \end{aligned}$$

The overdamped case consists of two exponential terms with two different decay constants, viz $(\gamma _{\mathrm{d}}-|\delta _{\mathrm{d}}|)$ and $(\gamma _{\mathrm{d}}+|\delta _{\mathrm{d}}|)$. The oscillator returns to the equilibrium state without oscillating. Depending on the values of $\gamma _{\mathrm{d}}$ and $\delta _{\mathrm{d}}$, the normal mode can be either overdamped unstable or overdamped stable (see Fig. 2).
